# Insurance decisions under nonperformance risk and ambiguity

**DOI:** 10.1007/s11166-021-09364-7

**Published:** 2021-11-26

**Authors:** Timo R. Lambregts, Paul van Bruggen, Han Bleichrodt

**Affiliations:** 1 Erasmus School of Health Policy & Management, Erasmus University Rotterdam, Burgemeester Oudlaan 50, 3062 PA Rotterdam, Netherlands; 2 Department of Economics, Tilburg University, Tilburg, Netherlands; 3 Erasmus School of Economics, Erasmus University Rotterdam, Rotterdam, Netherlands; 4 CPB Netherlands Bureau of Economic Policy Analysis, The Hague, Netherlands; 5 Research School of Economics, Australian National University, Canberra, Australia

**Keywords:** Ambiguity, Insurance, Nonperformance risk, Prudence, C91 D81

## Abstract

An important societal problem is that people underinsure against risks that are unlikely or occur in the far future, such as natural disasters and long-term care needs. One explanation is that uncertainty about the risk of non-reimbursement induces ambiguity averse and risk prudent decision makers to take out less insurance. We set up an insurance experiment to test this explanation. Consistent with the theoretical predictions, we find that the demand for insurance is lower when the nonperformance risk is ambiguous than when it is known and when decision makers are risk prudent. We cannot attribute the lower take-up of insurance to our measure of ambiguity aversion, probably because ambiguity attitudes are richer than aversion alone.

**Supplementary Information:**

The online version contains supplementary material available at 10.1007/s11166-021-09364-7.

## Introduction

The motivation for this paper is an important puzzle in insurance economics: why do people take out too little insurance against risks with potential huge consequences, such as natural disasters and long-term care needs. Standard insurance theory suggests that such insurance should be valuable as it protects individuals against the potentially devastating costs of these events. In practice, however, the holding of such insurance is (too) low.^1^ Although various reasons have been put forward to explain this puzzle, it is still only partially understood. A better understanding is important for policy, as it may protect people from financial distress and governments from footing the bill.

One possible reason is that people are concerned that insurers will not pay out future claims. This is not unheard of. For example, after Hurricane Katrina, insurers denied coverage to people who had home insurance, but no additional flood coverage (Kunreuther & Pauly 2006). During the COVID-19 pandemic, insurers across the globe have been hesitant to pay out claims for business interruption insurance and there is a fair amount of ongoing litigation about whether lost business income due to lockdowns is covered or not. Concerns about such nonperformance may be particularly grave when benefits occur in the far future, which carries the risk that insurers may go bankrupt, and which makes the value of insurance inherently more risky and ambiguous.^2^,^3^ The purpose of this paper is to explore the role of ambiguity regarding nonperformance on insurance take-up. We consider the case of full insurance. Because full insurance is equivalent to what Ehrlich and Becker (1972) call self-protection, our results also help to better understand underinvestment in prevention.

Multiple theoretical predictions relevant to insurance with nonperformance risk pointed to the importance of (higher order) risk and ambiguity attitudes in explaining behavior.^4^ While the effect of risk aversion is equivocal (Dionne & Eeckhoudt, 1985), Peter & Ying (2020) show that ambiguity averse decision makers will reduce their demand for insurance when the nonperformance risk is ambiguous. Moreover, developments in the domain of higher order risk preferences, which relate to how people prefer to combine risks, suggest that risk prudence has an important effect: it decreases the demand for insurance with nonperformance risk (Eeckhoudt & Gollier, 2005). These theoretical predictions have, however, received little attention in the empirical literature.

We set up an experiment and relate uptake decisions for full insurance to both ambiguity and (higher order) risk and ambiguity preferences. Our main finding is that ambiguity of the nonperformance risk indeed decreases the demand for insurance. Risk attitudes are important in explaining insurance behavior: risk aversion increases insurance demand, while risk prudence, as predicted, affects it negatively. We could not link the observed insurance behavior to our measure of ambiguity aversion. The reason was that the ambiguity attitudes we observed were richer than aversion alone: for more likely events our subjects were predominantly ambiguity loving (remember that insurance decisions involve losses). The main factor influencing insurance demand is the size of the insurable risk. The larger this probability is, the more likely our subjects were to demand insurance. While our findings on ambiguity attitudes are consistent with prospect theory, the findings on the role of the loss probability are not. They pose a challenge to prospect theory.

This paper proceeds as follows. Section 2 provides a review of the literature. Section 3 presents our experiment design. Section 4 presents our empirical findings. Implications of which are discussed in Sect﻿ion 5. Section 6 concludes.

## Background

### Theory

Consider a standard full insurance policy. This theoretical product fully reimburses a loss $L$ occurring with probability $p$ (the *insurable risk*) and is available at an actuarially fair premium $\pi =pL$. In practice, however, insurance has a probability of nonperformance with which insurers do not pay out a valid claim. Nonperformance may occur for various reasons, one of which is that that benefits occur in the far future and the insurance company may no longer exist.

**Fig. 1:**
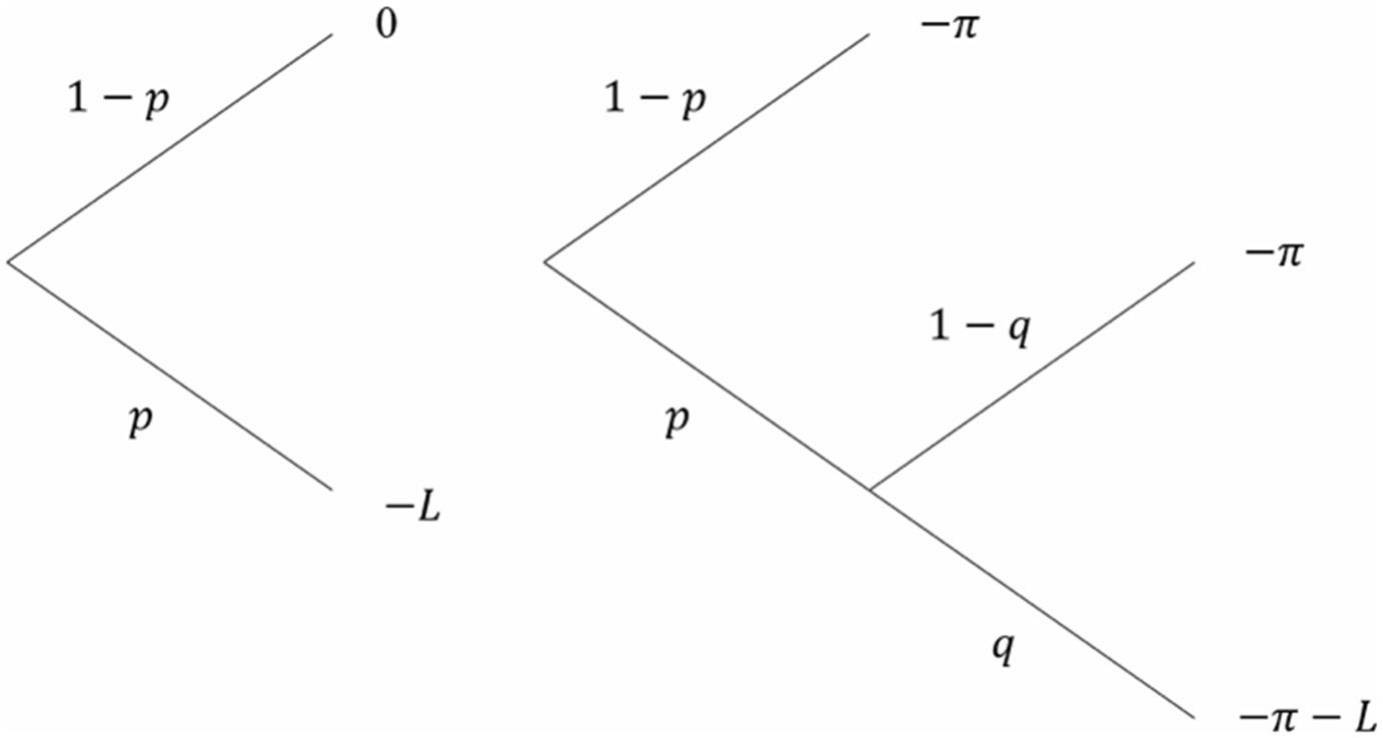
The insurance choice: no insurance (left) versus insurance with nonperformance risk (right)

If there is a risk of nonperformance $q$, buying insurance no longer eliminates the insurable risk, but reduces it from $p$ to $pq>0$. In our experiment, $p$ and $q$ may or may not be objectively known. If either $p$ or ﻿$q$ is unknown (or if both are), the decision is made under ambiguity. An individual’s choice whether to buy insurance can be schematically depicted as in Fig. 1.In the special case of full insurance, insurance with nonperformance risk is equivalent to Ehrlich and Becker's (1972) concept of self-protection (also called prevention): both reduce the risk of incurring a loss, but they do not completely remove it.^5^ Hence, while part of the literature that we discuss below is framed as prevention, their results are equally applicable to insurance decisions with a risk of nonperformance in the special case of full insurance. One caveat that should be made here is that in Fig. 1 the choice is binary: either insurance or no insurance. Most of the literature on self-protection is about the level of effort. Denuit et al. (2016) show, however, that the same difficulties that have been identified to choice of the optimal level of self-protection apply to the binary choice between two levels of self-protection.

The literature has identified several factors that affect demand for insurance with nonperformance risk. The first contributions focused on the role of risk aversion, taking $q$, the probability of nonperformance, as known. Dionne and Eeckhoudt (1985) show that for expected utility maximizers with a quadratic utility function, risk aversion increases (decreases) the uptake of insurance with nonperformance risk when $p<0.5$ ($p>0.5$). This result arises because the insurance itself is risky; purchasing it deteriorates the worst possible outcome with the insurance premium paid (Bryis & Schlesinger, 1990).^6^ Jullien et al. (1999) extend Dionne and Eeckhoudt’s (1985) analysis to more general utility functions. They derive that in general the effect of risk aversion on insurance uptake is also inverse U-shaped: risk aversion increases insurance uptake up to some endogenous threshold of $p$, which depends on the utility functions of both agents under comparison, and then decreases it.^7^

Eeckhoudt and Gollier (2005) show that higher order risk attitudes also affect the demand for insurance. In particular, they prove that, compared with the risk-neutral benchmark, risk prudence reduces the demand for insurance. Peter (2020) shows that this also holds when the benchmark agents has more general risk preferences. Risk prudence implies an aversion to downside risk or to combining bad events with bad events (Eeckhoudt & Schlesinger, 2006). Buying insurance with nonperformance risk entails more downside risk, as two bad events can occur simultaneously: paying the premium, while also incurring the loss. This makes such insurance unattractive to risk prudent individuals. Menegatti (2009) and Peter (2017) extend these findings to an intertemporal model where the decision maker pays an insurance premium now to cover a future loss in the presence of nonperformance risk. Courbage and Rey (2006) extend the analysis of the effect of prudence on insurance to decisions involving both health and wealth. They show that individuals who lose more from being sick will demand more insurance, provided that they are less prudent about wealth.

The above analyses are based on expected utility. Baillon et al. (2020) consider rank-dependent utility (prospect theory for losses) and derive the implications of probability weighting on prevention, which, as we noted above, is equivalent to full insurance with nonperformance risk. They show that for intermediate probabilities, inverse S-shaped probability weighting, the most commonly observed case, will lead to underinsurance.

Finally, Snow (2011) and Peter and Ying (2020) study the impact of ambiguity aversion when the insurance decision is made under ambiguity. They both assume that the decision maker is risk and ambiguity averse. This assumption is not uncontroversial as people tend to be ambiguity seeking for unlikely events and losses (Kocher et al., 2018), which typically occur in insurance decisions. Peter and Ying (2020) show that an ambiguous nonperformance risk always reduces the demand for insurance (compared with a known nonperformance risk), regardless of whether the insurable risk is ambiguous or not. Snow (2011) shows that an ambiguous insurable risk increases the demand for insurance in the presence of a known nonperformance risk.^8^ This happens because insurance reduces the ambiguity of the insurable risk and an ambiguity averse decision maker likes reductions in ambiguity.^9^

Table 1 summarizes the various cases. The first letter indicates whether the insurable risk ($p$) is *known* ($K$) or *unknown* ($U$) and the second letter whether the nonperformance risk ($q$) is known ($K$) or unknown ($U$). A $+$ ($-$) sign indicates that insurance demand is higher (lower) for the row combination than for the column combination. A question mark indicates that this case has not yet been explored in the literature. Combining the results of Peter and Ying (2020) and Snow (2011), Table 1 shows that the demand for insurance must be higher when the insurable risk is unknown and the nonperformance risk is known than when the insurable risk is known and the nonperformance risk is unknown: in the first case, insurance reduces ambiguity, whereas in the latter case, it increases it.^10^

**Table 1:** Relative attractiveness of insurance with nonperformance risk under risk and ambiguity (row vs column)

Treatment	*KK*	*KU*	*UK*
$KU$	$-$ ^a^		
$UK$	$+$ ^b^	$+$ ^c^	
$UU$	?	?	$-$ ^a^

Treatments *KK*, *KU*, *UK* and *UU* indicate whether the insurable risk ($p$ ) and nonperformance risk ($q$ ) respectively are known ($K$ ) or unknown ($U$). Superscripts indicate whether the prediction is derived from Peter and Ying (2020) (^a^), Snow (2011) (^b^) or inferred from both (^c^)

The impact of higher order ambiguity preferences has hardly been explored. This is partly because they were defined only recently (Baillon, 2017). Peter and Ying (2020) derive that more ambiguity leads to less insurance demand provided that ambiguity prudence is not too large.

### Empirical evidence

Several studies have shown that the introduction of a known nonperformance risk decreases the demand for full insurance (Herrero et al., 2006; Wakker et al., 1997; Zimmer et al., 2009, 2018). Bajtelsmit et al. (2015) show that this also holds when the nonperformance risk is ambiguous. Biener, Landman and Santana (2019) find tentative evidence that an ambiguous nonperformance risk may reduce insurance demand compared to a known one. This is an empirical matter that we will further address in our current study.

Krieger and Mayrhofer (2017) and Masuda and Lee (2019) investigate the role of higher-order risk preferences in prevention decisions. Krieger and Mayrhofer (2017) find that more prudent decision makers invest less in prevention, which is consistent with the predictions of Eeckhoudt and Gollier (2005). Masuda and Lee (2019) also find that their subjects exert too little preventive effort regardless of the timing of the loss. They argue that their results cannot be explained by expected utility and suggest probability weighting as an alternative explanation, in line with the analysis of Baillon et al. (2020).

## Experiment

The purpose of our paper is twofold. First, we empirically test the theoretical predictions in Table 1 and provide a complete picture of the effects of unknown insurable and nonperformance risks on the demand for insurance. Second, we explore whether the demand for insurance can be related to risk aversion and prudence and, in the cases where the risks are unknown, to ambiguity aversion and prudence. We therefore consider both known and unknown insurable risk and nonperformance risk: we consider each of the four cases $KK$(both the probability of the insurable loss and the probability of nonperformance are known), $KU$, $UK$, and $UU$.

### Subjects

117 students from Erasmus University Rotterdam participated in the experiment, which was conducted at the Erasmus Behavioural Lab (EBL). There were 12 sessions with a maximum of 12 participants per session. Participants were seated in cubicles to avoid interaction. They were recruited from the subject pool of the EBL and they were instructed that the experiment could last up to 1 h and 15 min. Participants were told that their expected payoff from the experiment was €25 with a minimum of €3.40 and a maximum of €134.20. Before starting the experiment, participants received €25 in cash. They were told that the experiment involved both the possibility of losing money and the possibility of gaining money and that they could pay eventual losses out of the €25. In this way, participants were stimulated to think of the average €25 payment as a reference point. The average payment per participant turned out to be €23.90.

### Incentives

The experiment was incentivized using the PRINCE incentive system (Johnson et al. 2021), which has the advantage of making incentive compatibility transparent to the participants. The main property of PRINCE is that the choice to be played out for real is chosen upfront. In our study, prior to the experiment, participants were asked to pick any of 92 sealed envelopes representing the 92 choice tasks in the experiment. The participants took their selected envelope to their cubicle, making it clear that their answers could not affect the selection of the task that they would play out for real. They were not allowed to open their envelope until they returned to the instruction room after the experiment. The experiment choices were framed as instructions to the experimenters: for all 92 choice tasks participants were asked: “If your envelope contains this choice, which option would you like us to play out for real?” The choice tasks that the envelope contained described the entire choice task (that is, both options from a given task). The option that was played out for real was the one chosen by the participant in that choice task.

### Experiment design

The experiment consisted of two parts, one measuring the demand for insurance with nonperformance risk, the other measuring participants’ attitudes towards risk and ambiguity. In total, the experiment consisted of 92 binary choices: 56 choices measuring the demand for insurance, 32 choices measuring risk and ambiguity attitudes, and 4 choices that were repeated to test the quality of the data. A complete list of the choices is presented in Tables 4–9 in Online Appendix A.

#### Insurance tasks

We used four treatments to measure the demand for insurance with nonperformance risk, which varied depending on whether the insurable risk and the nonperformance risk were known ($K$) or unknown ($U$). Thus, we studied all situations $KK$, $KU$,$UK$ and $UU$ listed in Table 1. There were 9 choices per treatment. In addition, we included five choices per treatment involving gains to make sure the expected payment from the experiment was €25. These choices are not used in the analyses.

**Fig. 2:**
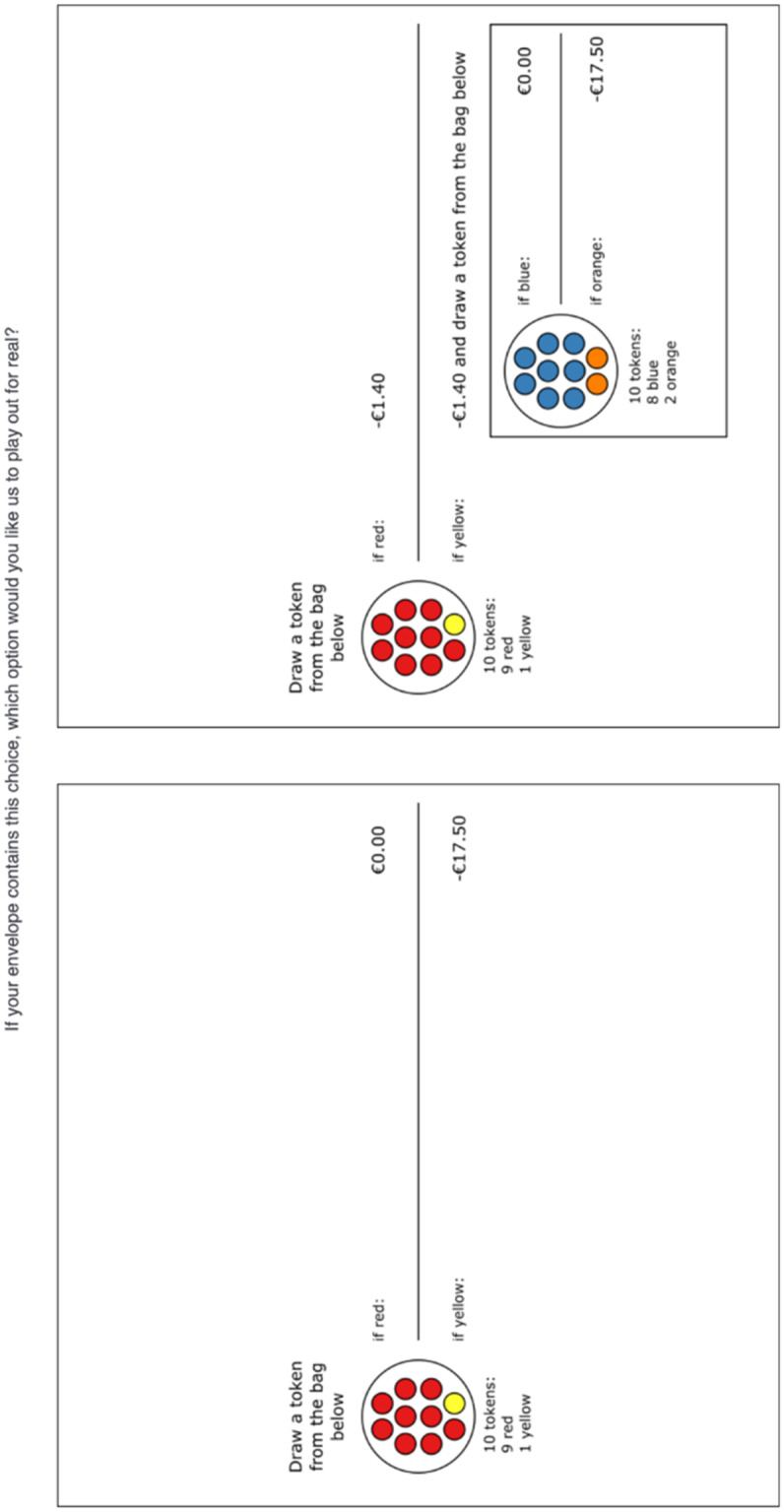
Example of a choice task with known probabilities

**Fig. 3:**
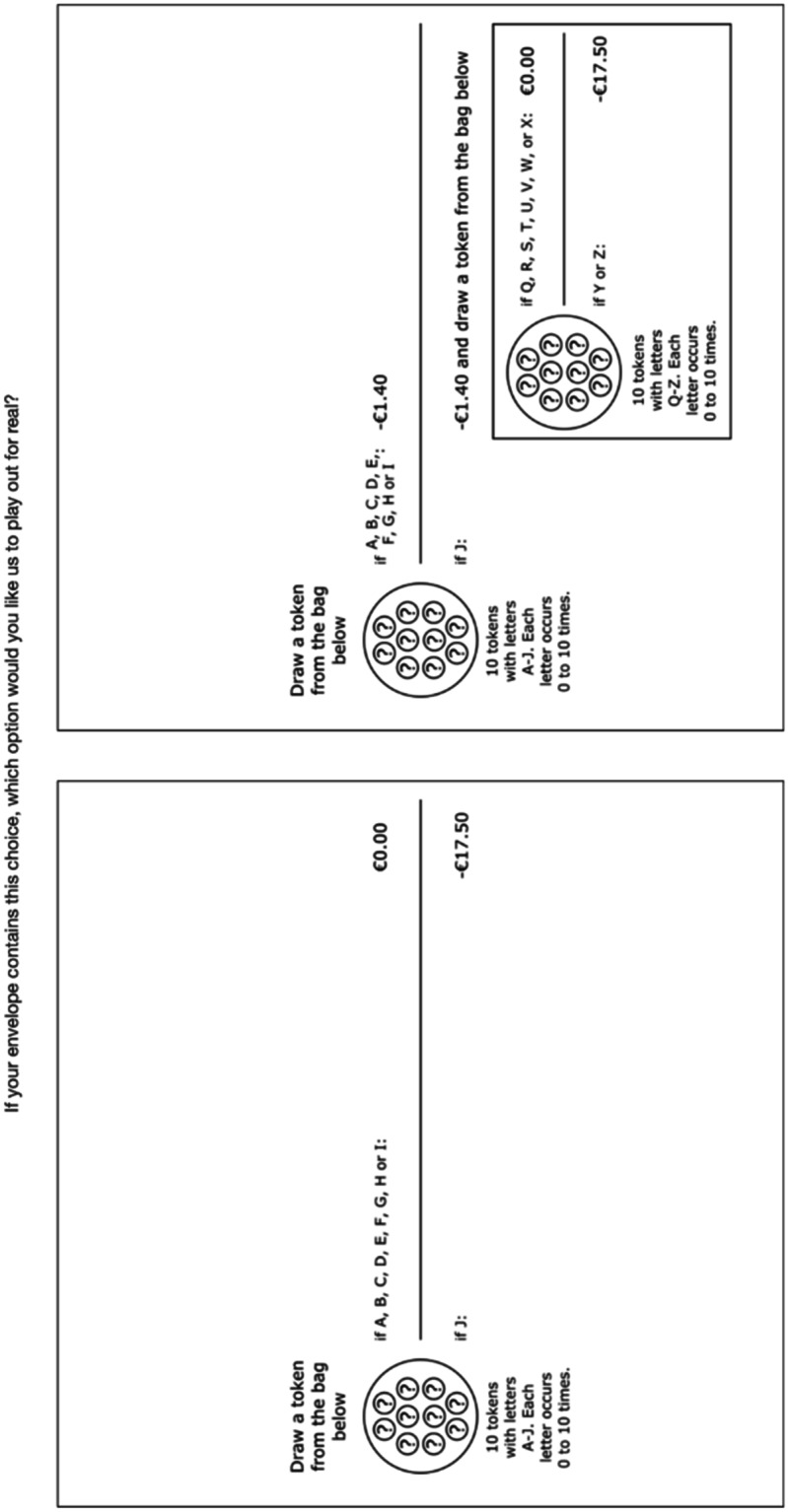
Example of a choice task with unknown probabilities

Fi﻿gure 2 shows an example of a choice from treatment $KK$, Fig. 3 shows the counterpart from treatment $UU$. All choices were represented as situations where a token is drawn from a bag, possibly followed by a second draw from another bag. All bags contained 10 tokens. For risky choices, the tokens were colored (red and yellow or blue and orange as in Fig. 2), for ambiguous choices, they had a question mark (see Fig. 3). It was explained that the ambiguous bags contained 10 tokens with letters (A – J or Q – Z) in unknown proportion and that each letter could occur between 0 and 10 times. The bags were filled according to the instructions of a random person not affiliated with the experiment and participants were informed about this. To avoid suspicion, we asked each participant before the start of the incentivized choice tasks whether they wanted the letter A to be associated with the best outcome, the letter B with the second best outcome etcetera, or whether they wanted the ranking to be reversed (such that A was associated with the worst outcome and Z with the best outcome).

Figures 2 and 3 show that in the tasks measuring insurance demand, one option resembled no insurance and the other resembled insurance with a nonperformance risk. The no insurance option (we did not use this term in the experiment) involved a possible loss (€17.50 in Figs. 2 and 3). In the insurance option, subjects paid an actuarially fair premium (€1.40 in Figs. 2 and 3) to reduce the probability of the loss, but there was a nonperformance risk. This is depicted by the bag containing orange and blue tokens in Fig. 2 and by the bag containing tokens with letters Q – Z in Fig. 3. Note that we deliberately presented the insurance options as compound lotteries, to emphasize the differences in potential outcomes with the no insurance option.

#### Risk and ambiguity attitudes

The second part measured risk aversion (9 choices), risk prudence (9 choices), ambiguity aversion (9 choices), and ambiguity prudence (5 choices). Figures 10–13 in Online Appendix A give examples for each of these tasks. We used fewer choices to measure ambiguity prudence, because these questions were more complex and cognitively demanding. We developed these choice tasks to stay as close as possible to the insurance tasks. Hence, all tests involved only losses and €0. In addition, the risk and ambiguity prudence tasks were also presented in a compound form.^11^

A decision maker is *risk averse* when preferring a lottery over a mean-preserving spread of that lottery. To avoid the certainty effect, we chose to have both options risky. Then, risk aversion is defined as the preference:

$$(p: -k, 1-p:-r)\succsim \left(p: -k-\frac{1-p}{p}r, 1-p:0\right)$$

for all $p\in [\mathrm{0,1}]$ and $k,r>0$. In the left lottery the harms $-k$ and $-r$ are disaggregated, i.e., only one of them occurs, while in the right lottery they are aggregated. In our experiment, $p$ varied from $0.1\;{\mathrm{to}}\;0.9$  in steps of $0.1$.

Eeckhoudt and Schlesinger (2006) define *risk prudence* as the preference of $(0.5:-k,0.5: \tilde{\varepsilon })$ over $(0.5:-k+\tilde{\varepsilon }, 0.5:0)$ for all $k>0$ and for all zero mean random variables $\tilde{\varepsilon }$. This can be interpreted as a preference to disaggregate the two harms $-k$ and $\tilde{\varepsilon }$ over aggregating them.^12^ The decision maker rather bears the loss in the state of the world where they do not bear the risk. In our experiment, $\tilde{\varepsilon }$ is a binary random variable with equiprobable outcomes $s$ and $-s$. For each task, a sure amount *c* was deducted such that all possible outcomes were in the loss domain and choices could not be affected by loss aversion.

Where risk aversion and risk prudence are conditions about the spread of outcomes over different states, ambiguity aversion and ambiguity prudence are conditions about the spread in probabilities. Let $\tilde{\varepsilon }$ now denote a zero-mean nondegenerate random variable to which a probability $p$ can be added such that $p+\tilde{\varepsilon }\in [\mathrm{0,1}]$. The lotteries $(p:-k,1-p:0)$ and $(p+\tilde{\varepsilon }:-k,1-p-\tilde{\varepsilon }:0)$ then have the same expected probabilities of losing $k$, but for the first lottery this probability is known, while for the second lottery it is unknown. A decision maker is *ambiguity averse* if they prefer the lottery with known probabilities over the lottery with unknown probabilities:

$$(p:-k,1-p:0)\succsim (p+\tilde{\varepsilon }:-k,1-p-\tilde{\varepsilon }:0)$$

for all zero mean random variables $\tilde{\varepsilon }$, for all $p+\tilde{\varepsilon }\in[\mathrm{0,1}]$ and for all $k$.

Baillon (2017) defined the notion of ambiguity prudence. To define ambiguity prudence, we change the notation slightly. Let $(\{p,q+\tilde{\varepsilon }\}:-L)$ denote a lottery that determines with an unknown probability whether a loss of $L>0$ is given with known probability $p$ or with unknown probability $q+\tilde{\varepsilon }$ and else nothing happens (i.e., the outcome is 0). Now consider a given increase $k$ in the probability of the loss $L$. Ambiguity prudence says that the decision maker will prefer to disaggregate these two harms. In other words, ambiguity prudent decision makers will prefer $(\{p+k,q+\tilde{\varepsilon }\}:-L)$ to $(\{p,q+\tilde{\varepsilon }+k\}:-L)$.^13^ As an example, we have $p=0.5$, $q=0.35$ and $k=0.4$ in Fig. 10 in the Online Appendix A.

Participants randomly started with the insurance part or with the risk and ambiguity part of the experiment. The order of the sub-parts within these parts and of the choice tasks within these sub-parts was also randomized.^14^ The location (left or right) of any two options was randomized for each decision for each subject. After answering the incentivized choice tasks, participants were asked to answer four background questions. Two of these asked for subjects’ gender and nationality (two of the main sources of variation in experimental samples). The other two were directly related to insurance uptake, asking subjects to indicate which insurance products (e.g., mobile phone insurance, legal aid insurance) they have and how large the voluntary deductible on their mandatory health insurance is.

Before the experiment, participants were given a generic instruction of the incentive structure in the instruction room (see Online Appendix B1). Further instructions for the choice tasks were provided upon starting the experiment and are shown in Online Appendix B2. After these instructions, participants answered four true–false questions to check their comprehension of the experiment. They could only proceed to the incentivized choice tasks once they had answered all comprehension questions correctly. In this way, participants received feedback on their understanding of the choice tasks. Additionally, summaries of the particularities of the choice tasks were given at the start of each sub-part and were followed by one true–false question. We ensured that an experimenter was available at all times to answer participants’ questions.

After the experiment, participants were asked to return to the instruction room and choices were played out for real. A token was drawn for each of the possible bags used in the experiment. After drawing the tokens, participants could open their envelopes and the choice task it contained was recreated. Risky probabilities were played out by drawing colored tokens from a bag. For each probability $p$, a token was drawn from a bag with a total of 10 red or yellow tokens. The bags with colored tokens were filled while the participants were present so that they could check that the bags contained the correct number of red and yellow tokens. Then, a token was drawn 9 times; that is for all probabilities $0.1\le p\le 0.9$ (Fig. 2 shows $p=0.1$). Similarly, for $q$, a token was drawn from a bag with a total of 10 blue or orange tokens in compositions representing $0.1\le q\le 0.9$ (Fig. 2 shows $q=0.2$). Ambiguous probabilities were played out by drawing a token from a bag containing letters A – J (for $p$) and from a bag containing letters Q – Z (for $q$).

## Results

### Risk and ambiguity attitudes

The theoretical results that we discussed in Section 2 show that risk and ambiguity attitudes play a central role in explaining insurance decisions with nonperformance risk. We, therefore, first present the results on risk aversion, risk prudence, ambiguity aversion, and ambiguity prudence before discussing our main result, the effect of ambiguity on the demand for insurance.

**Fig. 4:**
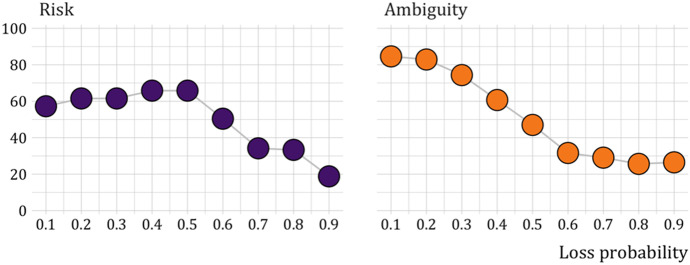
Proportion of risk averse and ambiguity averse choices for different loss probabilities

Figure 4 shows the proportion of risk and ambiguity averse choices split out by the loss probability. In the case of ambiguity, the displayed probability is the proportion of letters associated with the worst outcome. The figure shows evidence for the loss part of the fourfold pattern of risk and ambiguity aversion suggested by prospect theory: for small probabilities, subjects were mostly risk and ambiguity averse, for larger probabilities they were mostly risk and ambiguity seeking.

Overall, neither risk nor ambiguity aversion dominates. Bayesian tests provide strong support for the null that overall subjects are equally likely to choose the risky and the less risky option (Bayes factor^15^ ($BF$) $=0.08$) and support for the null that they are equally likely to choose the ambiguous and the unambiguous option ($BF=0.11$). However, as we noted, this is driven by a consistent aggregate pattern of risk and ambiguity aversion for small loss probabilities and risk and ambiguity loving choices for medium and large loss probabilities. Bayesian testing shows support for the hypothesis that the proportion of risk averse choices is correlated with the loss probability ($BF=5.7$) and very strong support for the hypothesis that the proportion of ambiguity averse choices is correlated with the loss probability ($BF=50.9$).

Individual tests show support for risk aversion for probabilities less than $0.6$ (all $BF>4.38$ except for probability $0.1$ for which the Bayesian test is inconclusive ($BF=0.72$), support for risk neutrality for probability $0.6$ ($BF=0.23$), and very strong support for risk seeking for probabilities exceeding $0.6$ (all $BF>62.5$). They also show very strong support for ambiguity aversion for probabilities less than $0.4$ (all $BF>2.03\times {10}^{5}$, for probability $0.4$ the test is inconclusive ($BF=2.86$)), support for ambiguity neutrality for probability $0.5$ ($BF=0.27$), and very strong support for ambiguity seeking for probabilities exceeding $0.5$ (all $BF>472.8$).

We further investigate subjects’ risk and ambiguity attitudes by classifying them into four different types: averse, loving, inverse S-shaped, and S-shaped. Inverse S-shaped is the assumption underlying prospect theory. It involves aversion for small probabilities of a loss but loving for large probabilities. S-shaped is the opposite, loving for small loss probabilities but aversion for large probabilities.

Subjects were classified as risk [ambiguity] averse if they chose the least risky [ambiguous] option at least twice both in the three tasks where the probability of a loss was at most $0.3$ (‘small probabilities’) and in the three tasks where the probability was at least $0.7$ (‘large probabilities’). They were classified as risk [ambiguity] loving if they chose the most risky [ambiguous] option at least twice in both the small and the large probability tasks. Subjects were classified as inverse S-shaped if they chose the least risky [ambiguous] option at least twice in the small probability tasks and no more than once in the large probability tasks. Finally, they were classified as S-shaped if they chose the riskiest option at least twice in the small probability tasks and no more than once in the large probability tasks.

**Fig. 5:**
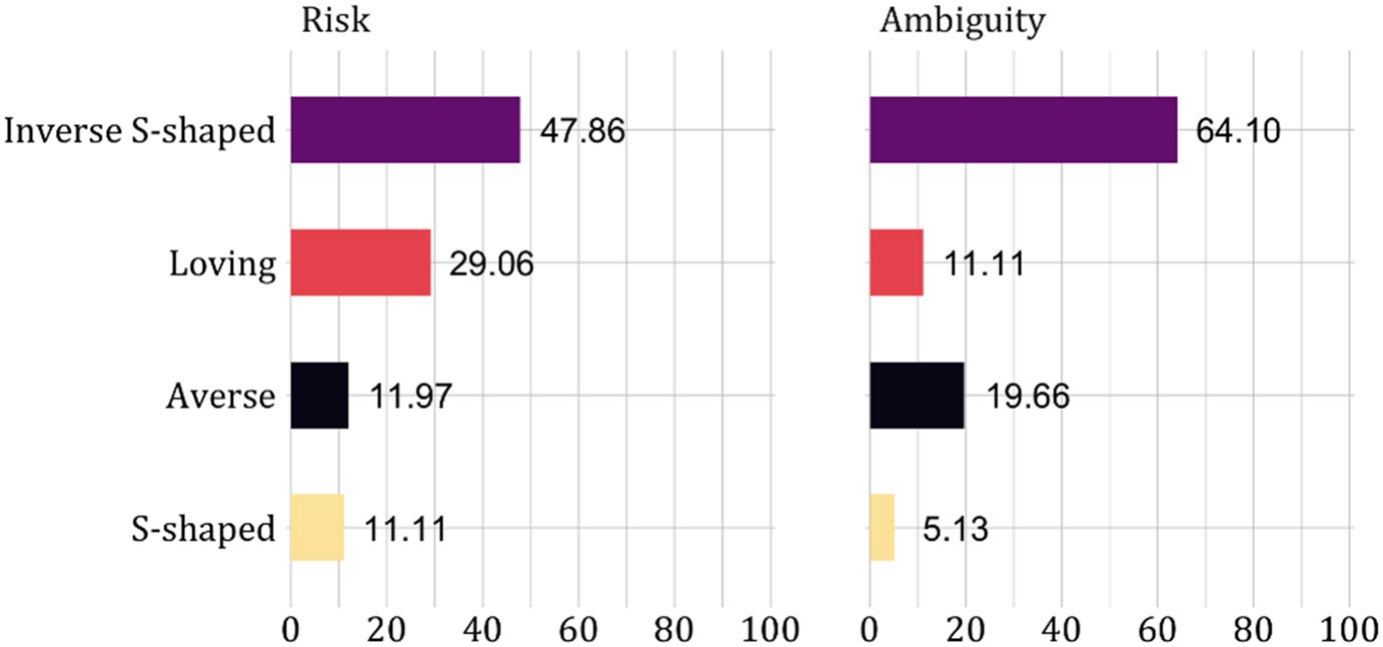
Proportion of subjects by risk and ambiguity attitudes

Figure 5 shows the classification of subjects for both risk and ambiguity. Inverse S is clearly the most common pattern. This is consistent with common findings (for risk, see e.g., Abdellaoui (2000) and Etchart-Vincent (2004), for ambiguity see e.g., Viscusi and Chesson (1999) and Baillon and Bleichrodt (2015)). Only a minority of the subjects behaved in line with the theoretically common assumptions of risk and ambiguity aversion. There are even substantially more subjects who were risk loving than risk averse.

**Fig. 6:**
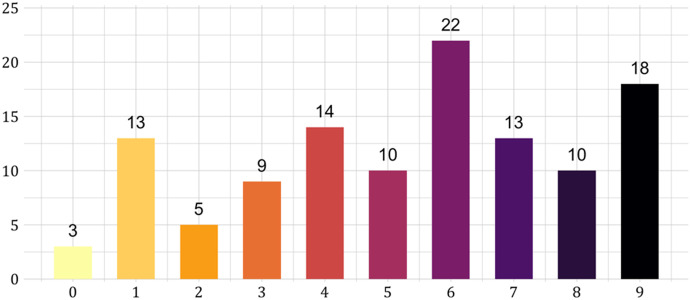
Number of subjects choosing each number of risk prudent choices

Figure 6 shows the subdivision of subjects depending on the number of risk prudent choices. The figure displays a tendency to risk prudence with on average ﻿$5.31$ risk prudent choices across the 9 tasks. A Bayesian test gives decisive support for risk prudence over the null of risk imprudence or neutrality ($BF = 3.51\times {10}^{6}$). This is inconsistent with the findings of Bleichrodt and van Bruggen (forthcoming), who found risk imprudence for losses.

**Fig. 7:**
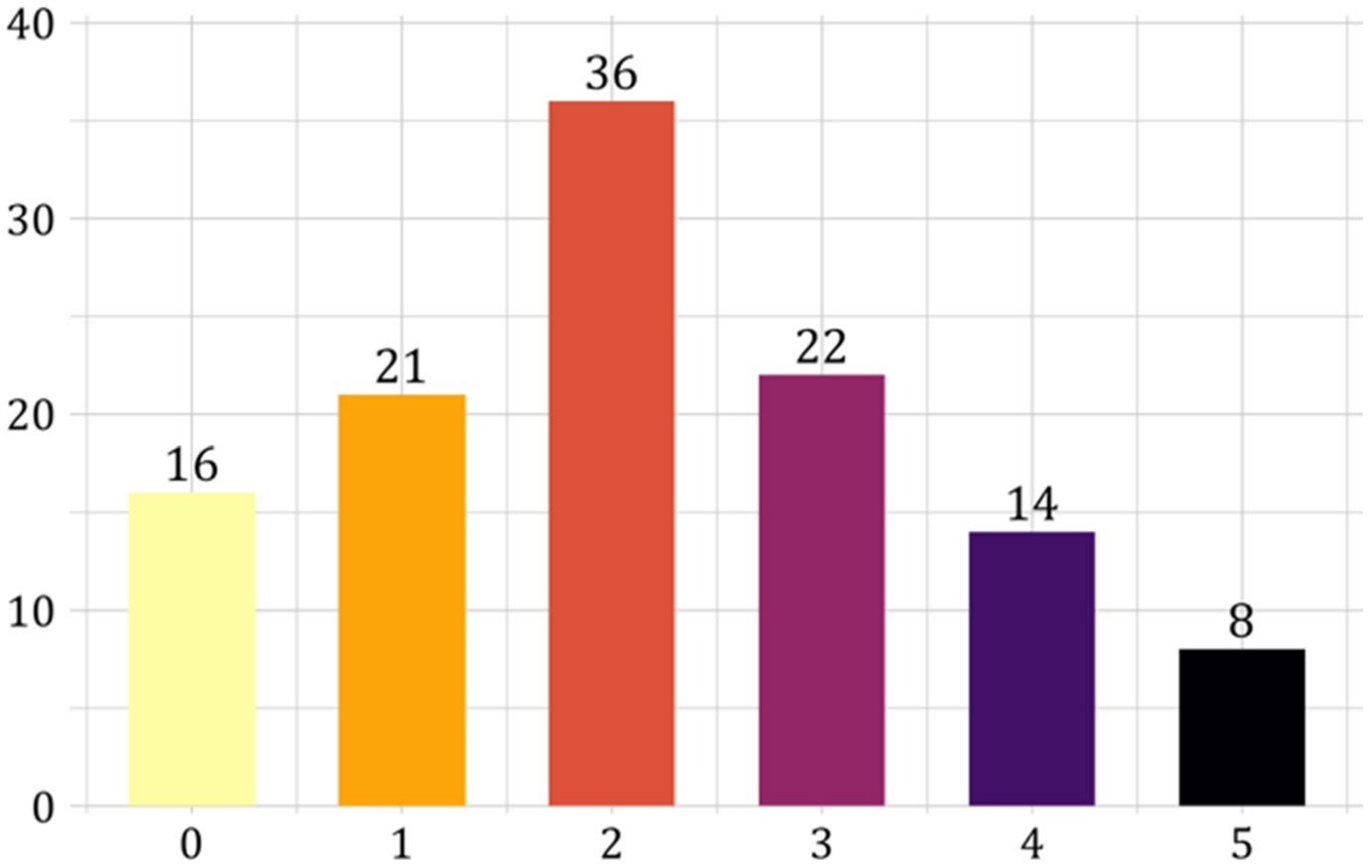
Number of subjects choosing each number of ambiguity prudent choices

Finally, Fig. 7 shows the subdivision of subjects depending on their number of ambiguity prudent choices. There is a slight tilt towards ambiguity imprudence. A Bayesian test shows support for the ambiguity imprudence over ambiguity prudence or neutrality ($BF = 24.13$). This finding goes against Baillon et al. (2018) who found predominant ambiguity prudence. However, they used gains whereas we use losses, which might explain the difference as this is consistent with a reflection effect for higher order preferences (Bleichrodt & van Bruggen, forthcoming).

### Insurance choices

**Fig. 8:**
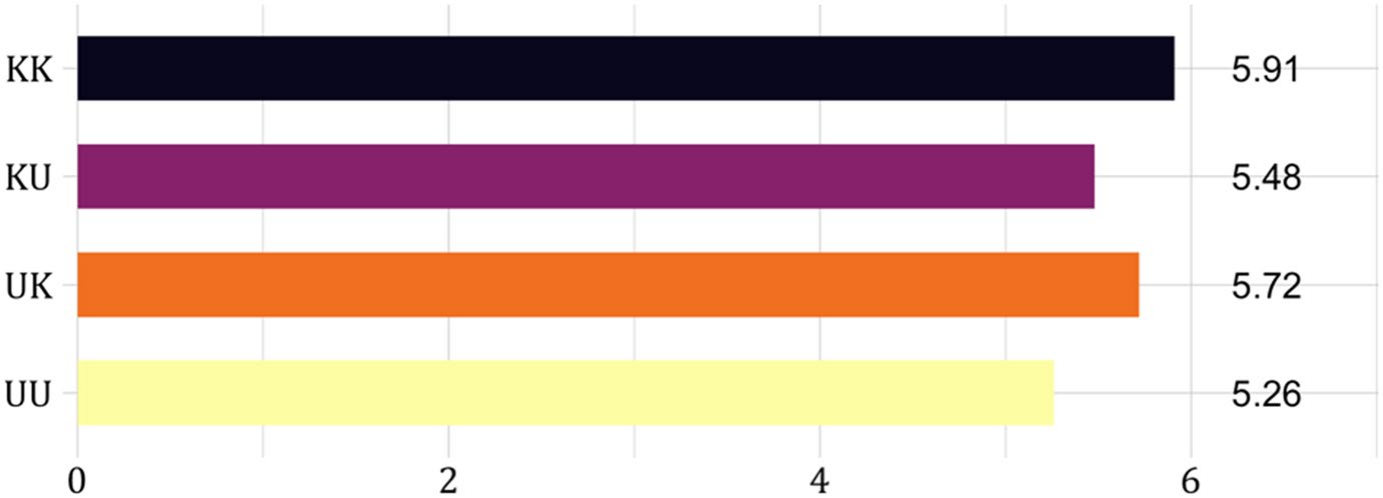
Mean number of insurance choices (out of 9) per treatment. *Notes:* Treatments $KK$, $KU$, $UK$ and $UU$ indicate whether the insurable risk and nonperformance risk respectively are known $(K)$ or unknown $(U)$

The central question of our paper is how unknown insurable risks and nonperformance risks change insurance decisions compared to the case where these risks are known. Figure 8 shows the mean number of choices (out of 9) in which subjects chose the insurance option. The results illustrate that moving from a known to an unknown probability made the insurance option less attractive. This effect is particularly pronounced for the probability of nonperformance. Bayesian testing shows support for the hypothesis that subjects choose insurance more often when the nonperformance risk is known than when it is unknown (treatments $KK$ and $UK$ versus treatments $KU$ and $UU$) ($BF=13.1$).^16^ However, when comparing choices for known and unknown insurable risks (treatments $KK$ and $KU$ versus treatments $UK$ and $UU$), we find support for the null that the number of insurance choices is the same ($BF=0.18$).

We obtain mixed results regarding the theoretical predictions outlined in Table 1. As mentioned in the previous paragraph, we find support for the prediction of Peter and Ying (2020) that an ambiguous nonperformance risk reduces the demand for insurance.^17^ However, we find no support for Snow’s (2011) prediction that insurance demand should be higher in treatment UK than in treatment KK. A Bayesian test supports the null that insurance demand was the same in these two treatments ($BF=0.13$). Similarly, we find no support for the prediction derived by combining the results of Peter and Ying (2020) and Snow (2011) that the demand for insurance should be higher in treatment UK than in treatment KU. A Bayesian test again supports the null that the demand for insurance was the same in these two treatments ($BF=0.18$).

It should be kept in mind though that the theoretical predictions of Peter and Ying (2020) and Snow (2011) are made under the assumption that subjects are risk and ambiguity averse. We saw in Sect. 4.1 that this is true for most subjects for probabilities less than 0.5. The probability of nonperformance was always less than 0.5 in our experiment, but the insurable risk could exceed $0.5$. An analysis of only those insurance choices in which the insurable risk was at most 0.4 confirmed all previous results with one important exception: Snow's (2011) prediction that subjects should be more inclined to choose insurance in treatment $UK$ than in treatment $KK$ was now very strongly supported ($BF=39.6$).^18^

**Fig. 9:**
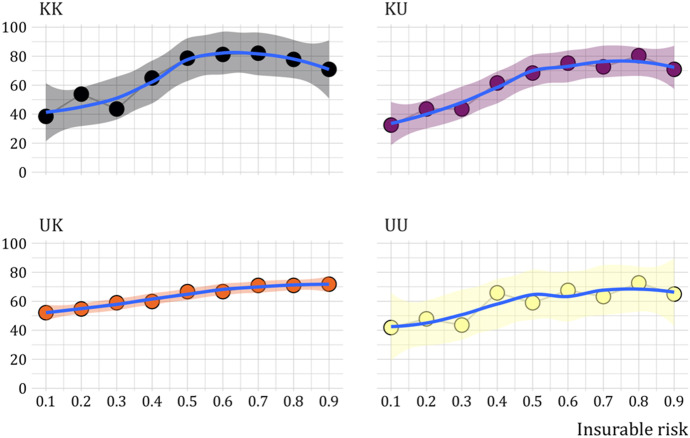
Proportion of insurance choices for each treatment by insurable risk. *Notes:* Treatments $KK$, $KU$, $UK$  and $UU$  indicate whether the insurable risk and nonperformance risk respectively are known $(K)$ or unknown $(U)$. Trend line fitted by loess method. Bands show the 95% confidence interval

Figure 9 shows for each of the four treatments how the proportion of insurance choices varies with the insurable risk. There is a trend for subjects to choose insurance more often when the insurable risk increases,^19^ particularly when it is known. Bayesian tests shows support for a positive correlation between the number of insurance choices and the insurable risk in all treatments (all $BF>4.8$, taking all treatments together the $BF=12.4$).

The dependence of insurance choice on the insurable risk is at odds with the predictions of inverse S-shaped weighting, the dominant pattern observed in our risk aversion tasks, if utility is linear and reduction of compound lotteries holds.^20^ Inverse S with linear utility predicts that subjects will be more inclined to buy insurance for small than for large loss probabilities, which is the opposite pattern of what we observe. The same pattern emerges when we restrict the analysis to those subjects who were actually classified as inverse S in the analysis of risk attitudes described above. The pattern of insurance choices is consistent with S-shaped weighting, but the number of subjects displaying S-shaped weighting is too low to perform meaningful analyses. The assumption of reduction of compound lotteries is not innocuous (e.g., Bernasconi, 1994). We therefore also analyzed the results under a recursive rank-dependent utility model, but this performed even worse (see Online Appendix D for details).

To extend our understanding of what drives the observed insurance decisions, we performed probit analyses with the choices in our insurance tasks as the dependent variables. In line with theoretical predictions, the results in Table 2 show that an unknown nonperformance risk leads to less insurance demand. On the other hand, ambiguity of the insurable risk has no effect on insurance demand. The lower demand for insurance with unknown nonperformance risk cannot be attributed to our measure of ambiguity aversion. Because ambiguity preferences cannot impact insurance decisions in treatment $KK$, where all risks were known, and because ambiguity aversion is predicted to affect demand differently for different treatments, we included interaction terms of ambiguity preferences and our treatments.^21^ None of the interaction terms is statistically significant. Ambiguity aversion has a positive impact only when it is included as a general variable for the subjects who had been classified as ambiguity averse (see Table 10 in Online Appendix E). However, this has no clear interpretation. Ambiguity prudence never affects the demand for insurance, but this should perhaps not come as a surprise given that its effect is not unequivocal as derived by Peter and Ying (2020).

**Table 2:** Probit regression results

	Choose insurance	Average marginal effect	Choose insurance	Average marginal effect
*KU*	-0.13** (0.05)	-0.05 (0.02)	-0.11 (0.09)	-0.04 (0.03)
*UK*	-0.06 (0.05)	-0.02 (0.02)	-0.06 (0.08)	-0.02 (0.03)
*UU*	-0.19*** (0.06)	-0.07 (0.02)	-0.17** (0.09)	-0.06 (0.03)
Risk aversion	0.07** (0.03)	0.02 (0.01)	0.07** (0.03)	0.02 (0.01)
Risk prudence	-0.04** (0.02)	-0.02 (0.01)	-0.04** (0.02)	-0.02 (0.01)
$p$	1.11*** (0.17)	0.40 (0.06)	1.11*** (0.17)	0.40 (0.06)
AA × *KU*			0.01 (0.13)	0.01 (0.05)
AA × *UK*			0.03 (0.12)	0.01 (0.04)
AA × *UU*			-0.03 (0.12)	-0.01 (0.04)
AP × *KU*			-0.08 (0.13)	-0.03 (0.05)
AP × *UK*			-0.05 (0.12)	-0.02 (0.04)
AP × *UU*			-0.02 (0.12)	-0.01 (0.04)
Male	0.23** (0.10)	0.08 (0.04)	0.23** (0.10)	0.08 (0.04)
Dutch	0.04 (0.10)	0.02 (0.04)	(0.04) (0.10)	0.02 (0.04)
Constant	-0.33 (0.21)		-0.34 (0.20)	

*N* = 4,176. Asterisks indicate a *p*-value < 0.05 (**) and < 0.01 (***). Robust standard errors between parentheses and clustered by subject. *KU*, *UK* and *UU* are treatment dummies indicating whether the insurable risk and nonperformance risk respectively are known (*K*) or unknown (*U*). Risk aversion and risk prudence are the number of risk averse and risk prudent choices (out of nine). AA is a dummy variable which takes the value of 1 if the subject chose the ambiguity averse option at least five times (out of nine). AP is a dummy variable which takes the value of 1 if the subject chose the ambiguity prudent option at least 3 times (out of 5). $p$ is the insurable risk and can take any decimal value from 0.1 till 0.9

Table 2 also shows that the insurable risk ($p$) has the largest marginal effect on insurance choice. It is positive, confirming that subjects were more inclined to choose the insurance option for higher loss probabilities. Subjects who were more risk averse chose insurance more often. The negative effects of risk prudence on insurance demand are in line with Eeckhoudt and Gollier (2005). Finally, we corrected for gender and nationality, the two main sources of variation in experimental studies.^22^ Males were more likely to choose insurance, but nationality had no effect.^23^,^24^

Because the theoretical models discussed in Section 2 concentrate on the risk and ambiguity averse subjects, we also separately analyzed choices with insurable loss probabilities of at most 0.5, the range for which most subjects were risk and ambiguity averse. Table 3 shows that the results change somewhat. In this range, the effect of an unknown nonperformance risk becomes less pronounced. On the other hand, the uptake of insurance with an unknown insurable risk and known nonperformance risk is higher and becomes significant, as predicted by Snow (2011). Also, the effect of risk attitudes becomes more important. As the results reported in Online Appendix E (Table 11; Fig. 14) suggest, consistent with the theoretical predictions of Jullien et al. (1999), this is because risk aversion only increases insurance demand up to a threshold of $p$.

**Table 3:** Probit regression results for insurable risks smaller than 0.5

	Choose insurance	Average marginal effect	Choose insurance	Average marginal effect
*KU*	-0.13* (0.08)	-0.05 (0.03)	-0.22* (0.12)	-0.08 (0.04)
*UK*	0.17** (0.09)	0.06 (0.03)	0.13 (0.12)	0.05 (0.04)
*UU*	-0.12 (0.08)	-0.04 (0.03)	-0.18 (0.12)	-0.06 (0.04)
Risk aversion	0.13*** (0.03)	0.05 (0.01)	0.14*** (0.03)	0.05 (0.01)
Risk prudence	-0.06** (0.03)	-0.02 (0.01)	-0.06** (0.03)	-0.02 (0.01)
$p$	1.71*** (0.27)	0.63 (0.10)	1.73*** (0.30)	0.63 (0.11)
AA × *KU*			0.29 (0.18)	0.10 (0.07)
AA × *UK*			0.13 (0.16)	0.05 (0.06)
AA × *UU*			0.25 (0.16)	0.09 (0.06)
AP × *KU*			-0.12 (0.19)	-0.04 (0.07)
AP × *UK*			-0.05 (0.17)	-0.02 (0.06)
AP × *UU*			0.12 (0.17)	0.05 (0.06)
Male	0.29** (0.13)	0.11 (0.05)	0.28** (0.13)	0.10 (0.05)
Dutch	0.05 (0.14)	0.02 (0.05)	0.05 (0.13)	0.02 (0.05)
Constant	-0.88*** (0.24)		-0.88*** (0.23)	

*N* = 1,856. Asterisks indicate a *p*-value < 0.10 (*), < 0.05 (**) and < 0.01 (***). Robust standard errors between parentheses and clustered by subject. *KU*, *UK* and *UU* are treatment dummies indicating whether the insurable risk and nonperformance risk respectively are known (*K*) or unknown (*U*). Risk aversion and risk prudence are the number of risk averse and risk prudent choices (out of nine). AA is a dummy variable which takes the value of 1 if the subject chose the ambiguity averse option at least five times (out of nine). AP is a dummy variable which takes the value of 1 if the subject chose the ambiguity prudent option at least 3 times (out of 5). $p$ is the insurable risk and can take any decimal value from 0.1 till 0.4

## Discussion

Our main conclusion is that an ambiguous nonperformance risk indeed leads to a reduction in insurance demand compared to a known nonperformance risk. Previous studies have already shown that nonperformance risk reduces the demand for insurance. We show that the more realistic case where the nonperformance risk is unknown further reduces the demand for insurance. This could help explain people’s reluctance to take up insurance against for example natural disasters or long-term care needs. Our results may also help to understand why people fail to undertake prevention measures, which is equivalent to the full insurance decisions that we – like previous experiments on nonperformance risk – examine.

Previous theoretical studies have primarily analyzed insurance decisions with nonperformance risk by assuming that decision makers are risk and ambiguity averse. If we restrict attention to the choices for which most subjects were risk or ambiguity averse, then most of these predictions were supported. Risk aversion leads to more insurance demand, while risk prudence reduces it, which is consistent with theoretical predictions (Dionne & Eeckhoudt, 1985; Eeckhoudt & Gollier, 2005). We can also confirm Peter and Ying's (2020) prediction that an ambiguous nonperformance risk leads to less insurance than a known nonperformance risk. At probabilities for which most individuals are risk and ambiguity averse, we found support for Snow's (2011) prediction that ambiguity of the insurable risk leads to more insurance demand.

Although we find that ambiguity of the nonperformance risk decreases the demand for insurance, ambiguity aversion did not appear to drive this effect in our regression analysis. In the analysis, ambiguity aversion was included as a dummy indicating whether a subject mostly chose the ambiguity averse option or not, consistent with the theoretical literature where ambiguity aversion is taken as a universal preference.^25^ Our data shows that the assumption of uniform risk and ambiguity aversion is too restrictive. Most subjects displayed the common empirical pattern of risk and ambiguity aversion for small loss probabilities and risk and ambiguity seeking for larger loss probabilities. A general ambiguity aversion variable cannot capture this diversity of ambiguity preferences within subjects and may therefore fail to fully pick up the effects of ambiguity averse (or seeking) preferences.

We find that most subjects are risk prudent, which goes against Bleichrodt and van Bruggen (forthcoming) who find clear evidence of risk imprudence for losses. The different findings may be due to differences in presentation: to ensure internal consistency with the presentation of the insurance tasks, the presentation of the prudence tasks in our experiment differed from the presentation in Bleichrodt and van Bruggen (forthcoming).^26^ We find evidence of ambiguity imprudence for losses, which is consistent with the reflection effect for higher order risk preferences observed by Bleichrodt and van Bruggen (forthcoming) and the predominant ambiguity prudence observed by Baillon, Schlesinger and van de Kuilen (2018) for gains.

The dependence of insurance choices on the insurable risk remains puzzling. Experimental research has long found (and been unable to explain) a similar dependency in actuarily fair insurance choices without nonperformance risk (Slovic et al., 1977). As we pointed out, this dependency is inconsistent with inverse S-shaped probability weighting if utility is linear. Usually empirical studies find that utility is close to linear for the stakes involved in our study. This poses the question about the external validity of elicited risk preferences: to what extent can they explain other choices people make? We are not alone in observing that inverse S-shaped probability weighting does not predict choice behavior well. Baillon et al. (2019) performed a field experiment in the Philippines in which they tried to nudge health behavior but did not observe the pattern predicted by inverse S-shaped weighting. Jaspersen et al. (forthcoming) explored to what extent models of decision under risk can predict insurance choices. While they found that these insurance choices were coherent and correlated with measures of risk attitude, the models they explored (which included expected utility and prospect theory) predicted these choices poorly and generally performed worse than simple heuristics.

Our results offer insights into the demand for long-term care insurance, which can benefit both policy makers and insurers. Uncertainty about the pay-out of future claims reduces insurance demand. Reducing such ambiguity could increase insurance uptake. This can be achieved through, for example, a common guarantee fund that insures against insurer bankruptcy. When such funds are already in place, increased awareness and transparency may further reduce ambiguity. The premium increase people would be willing to pay for such ambiguity reducing guarantees can be examined in future research. If needed, governments could support or subsidize such guarantees. Our study suggests that it is a worthwhile avenue to explore.

## Conclusion

An important policy puzzle is why people underinsure against uncertain losses that occur in the far future, such as long-term care needs. Our results show that one possible reason is the unknown nonperformance risk that comes with such insurance. Our results are largely consistent with the predictions made by theoretical models. An ambiguous nonperformance risk decreased the demand for insurance compared with a known nonperformance risk. Risk attitudes play an important role in explaining insurance demand: risk aversion increases insurance demand, while risk prudence reduces it. The effect of ambiguity attitudes was less clear, probably because they are richer than ambiguity aversion alone.

## Supplementary Information

Below is the link to the electronic supplementary material.

Supplementary file1 (PDF 1421 KB)

## Footnotes

1 See Kunreuther (1996) and Pestieau and Ponthière (2012) for a discussion of these respective underinsurance puzzles.

2 Li et al. (2020) make a similar point and observe that insolvency risk of insurers increases over time.

3 Although state guaranty associations exist in the US to protect against the insolvency of insurers, some states do not allow to advertise this fact, and many consumers are unaware of their existence.

4 Most predictions have been made in the context of self-protection. Given the equivalence to full insurance with nonperformance risk, we refer to these as insurance predictions throughout.

5 Buying insurance at premium $\pi =p(1-q)L$ is then a risk reducing activity equivalent to exerting preventive effort.

6 Doherty and Schlesinger (1990) show that a similar argument applies to partial insurance with a nonperformance risk, rendering the effect of risk aversion on insurance demand indeterminate.

7 Peter (2020) finds qualitatively similar results for an exogenous threshold that depends only on the utility of the benchmark agent.

8 Viscusi (1979) already noted that a more precise (i.e., less ambiguous) probability assessment conversely reduces insurance demand.

9 Alary et al. (2013) derive the opposite prediction when assuming that insuring (in their paper self-protection) increases the ambiguity of the insurable risk. When insuring does not affect ambiguity, ambiguity aversion may either decrease or increase insurance demand. Snow's (2011) assumption is more natural for our context, and matches our experimental implementation, hence we follow his prediction.

10 Denoting the demand for insurance given treatment $t$ as $\text{Dt}$, if $\text{D(UK)>D(KK)}$, which is what Snow (2011) shows, and $\text{D(KK)>D(KU)}$, which is what Peter and Ying (2020) show, then it logically follows that $\text{D(UK)>D(KU)}$.

11 Prudence can depend on the presentation of lotteries. Recent studies found more prudence for compound lotteries than for reduced lotteries (Deck & Schlessinger, 2018; Haering et al. 2020).

12 Of course, the lottery $\stackrel{\sim }{\varepsilon }$ is only seen as a harm if the decision maker is risk averse.

13 This definition implicitly assumes that all probabilities of a loss lie within the $[\mathrm{0,1}]$ interval.

14 However, the ambiguity prudence sub-part was always last within the risk and ambiguity part of the experiment, because of the complexity of those choice tasks.

15 The Bayes factor (BF) indicates how much more likely the alternative hypothesis is than the null. A BF larger than 3 is usually interpreted as providing some support for the alternative, a BF larger than 10 as providing strong support for the alternative, and a BF larger than 30 as providing very strong support for the alternative. Similarly a BF less than 0.33 is interpreted as providing some support for the null, less than 0.1 as providing strong support for the null, and less than 0.03 as providing very strong support for the null. BFs between 0.3 and 3 are interpreted as inconclusive evidence.

16 We tested this using the *contingencyBF* function in the R package BayesFactor (Morey & Rouder, 2018) with the assumption that the sampling type was joint multinomial.

17 However, if we separately compared treatments $KK$ and $KU$ and $UK$ and $UU$ the evidence was inconclusive ($BF=1.01$ and $BF=1.46$, respectively).

18 Most other tests were inconclusive.

19 This may sound trivial, but keep in mind that insurance was always actuarially fair, so the insurance premium also increased with the insurable risk. Jang and Hadar (1995) have shown that demand for actuarially fair insurance is not necessarily monotonic in the loss probability for a risk averse expected utility maximizer.

20 See Online Appendix C for a derivation.

21 Note that although ambiguity preferences should not play a role for decisions without ambiguity, risk preferences may play a role in decisions with or without ambiguity. Hence, there is no need to similarly interact risk preferences with our treatments.

22 None of these was associated with insurance choices in the experiment.

23 Excluding gender and nationality does not affect the interpretation of our results.

24 Dummies for the holding of different types of real-life insurance were not correlated with the insurance choices in our experiment. This is perhaps not surprising, as the sample consisted of students, who typically have few assets to insure.

25 Peter and Toquebeuf (2020) propose a framework that allows to formally derive results for ambiguity lovers, who often exhibit reversed behavior.

26 The difference was not due to the inclusion of the insurance task, as we found no difference in risk prudent choices between the subjects who started with the elicitation of risk and ambiguity attitudes and those who started with the insurance choices.

## References

[CR1] Abdellaoui M (2000). Parameter-free elicitation of utility and probability weighting Functions. Management Science.

[CR2] Alary D, Gollier C, Treich N (2013). The effect of ambiguity aversion on insurance and self-protection. The Economic Journal.

[CR3] Baillon A (2017). Prudence with respect to ambiguity. The Economic Journal.

[CR4] Baillon A, Bleichrodt H (2015). Testing ambiguity models through the measurement of probabilities for gains and losses. American Economic Journal: Microeconomics.

[CR5] Baillon A, Bleichrodt H, Emirmahmutoglu A, Jaspersen JG, Peter R (2020). Whenrisk perception gets in the way: Probability weighting and underprevention. Operations Research.

[CR6] Baillon, A., Capuno, J., O’Donnell, O., Tan, C., & van Wilgenburg, K. (2019). Persistent effects of temporary incentives: Evidence from a nationwide health insurance experiment. Tinbergen Institute Discussion Paper Series, 2019–078/V. 10.1016/j.jhealeco.2021.102580 34986436

[CR7] Baillon A, Schlesinger H, van de Kuilen G (2018). Measuring higher order ambiguity preferences. Experimental Economics.

[CR8] Bajtelsmit V, Coats JC, Thistle P (2015). The effect of ambiguity on risk management choices: An experimental study. Journal of Risk and Uncertainty.

[CR9] Bernasconi M (1994). Nonlinear preference and two-stage lotteries: Theories and evidence. The Economic Journal.

[CR10] Biener C, Landmann A, Santana M (2019). Contract nonperformance risk and uncertainty in insurance markets. Journal of Public Economics.

[CR11] Bleichrodt, H., & van Bruggen, P. (forthcoming). Reflection for higher order risk preferences. *Review of Economics and Statistics.*10.1162/rest_a_00980

[CR12] Bryis E, Schlesinger H (1990). Risk aversion and the propensities for self-insurance and self-protection. Southern Economic Journal.

[CR13] Courbage C, Rey B (2006). Prudence and optimal prevention for health risks. Health Economics.

[CR14] Deck C, Schlesinger H (2018). On the robustness of higher order risk preferences. Journal of Risk and Insurance.

[CR15] Denuit MM, Eeckhoudt L, Liu L, Meyer J (2016). Tradeoffs for downside risk-averse decision-makers and the self-protection decision. The Geneva Risk and Insurance Review.

[CR16] Dionne G, Eeckhoudt L (1985). Self-insurance, self-protection and increased risk aversion. Economics Letters.

[CR17] Doherty NA, Schlesinger H (1990). Rational insurance purchasing: Consideration of contract nonperformance. The Quarterly Journal of Economics.

[CR18] Eeckhoudt L, Gollier C (2005). The impact of prudence on optimal prevention. Economic Theory.

[CR19] Eeckhoudt L, Schlesinger H (2006). Putting risk in its proper place. American Economic Review.

[CR20] Ehrlich I, Becker G (1972). Market insurance, self-insurance, and self-protection. Journal of Political Economy.

[CR21] Etchart-Vincent N (2004). Is probability weighting sensitive to the magnitude of consequences? An experimental investigation on losses. Journal of Risk and Uncertainty.

[CR22] Haering A, Heinrich T, Mayrhofer T (2020). Exploring the consistency of higher order risk preferences. International Economic Review.

[CR23] Herrero C, Tomás J, Villar A (2006). Decision theories and probabilistic insurance: An experimental test. Spanish Economic Review.

[CR24] Jang YS, Hadar J (1995). A note on increased probability of loss and the demand for insurance. The Geneva Papers on Risk and Insurance Theory.

[CR25] Jaspersen, J.G., Ragin, M.A., & Sydnor, J.R. (forthcoming). Predicting insurance demand from risk attitudes. *Journal of Risk and Insurance.*

[CR26] Johnson CA, Baillon A, Bleichrodt H, Li Z, van Dolder D, Wakker PP (2021). Prince: An improved method for measuring incentivized preferences. Journal of Risk and Uncertainty.

[CR27] Jullien B, Salanié B, Salanié F (1999). Should more risk-averse agents exert more effort?. The Geneva Papers on Risk and Insurance Theory.

[CR28] Kocher M, Lahno A, Trautmann S (2018). Ambiguity aversion is not universal. European Economic Review.

[CR29] Krieger M, Mayrhofer T (2017). Prudence and prevention: An economic laboratory experiment. Applied Economics Letters.

[CR30] Kunreuther H (1996). Mitigating disaster losses through insurance. Journal of Risk and Uncertainty.

[CR31] Kunreuther H, Pauly M (2006). Rules rather than discretion: Lessons from Hurricane Katrina. Journal of Risk and Uncertainty.

[CR32] Li H, Neumuller S, Rothschild C (2020). Optimal annuitization with imperfect information about insolvency risk. Journal of Risk and Insurance.

[CR33] Masuda T, Lee E (2019). Higher order risk attitudes and prevention under different timings of loss. Experimental Economics.

[CR34] Menegatti M (2009). Optimal saving in the presence of two risks. Journal of Economics.

[CR35] Morey, R., & Rouder, J. (2018). *Bayesfactor: computation of Bayes factors for common**designs.* (version 0.9.12–4.2) [Computer software] from: CRAN.R-project.org/package=BayesFactor

[CR36] Pestieau P, Ponthière P, Costa-Font J, Courbage C (2012). Long-term care insurance puzzle. Financing long-term care in Europe.

[CR37] Peter R (2020). Who should exert more effort? Risk aversion, downside risk aversion and optimal prevention. Economic Theory.

[CR38] Peter R (2017). Optimal self-protection in two periods: On the role of endogenous saving. Journal of Economic Behavior and Organization.

[CR39] Peter, R., & Toquebeuf, P. (2020). Separating ambiguity and ambiguity attitude with mean-preserving capacities: Theory and applications. Working paper.

[CR40] Peter, R., & Ying, J. (2020). Do you trust your insurer? Ambiguity about contract nonperformance and optimal insurance demand. *Journal of Economic Behavior and **Organization*. 10.1016/j.jebo.2019.01.002

[CR41] Slovic P, Fischhoff B, Lichtenstein S, Corrigan B, Combs B (1977). Preference for insuring against probable small losses: Insurance implications. Journal of Risk and Insurance.

[CR42] Snow A (2011). Ambiguity aversion and the propensities for self-insurance and self-protection. Journal of Risk and Uncertainty.

[CR43] Viscusi WK (1979). Insurance and individual incentives in adaptive contexts. Econometrica.

[CR44] Viscusi WK, Chesson HW (1999). Hopes and fears: The conflicting effects of risk ambiguity. Theory and Decision.

[CR45] Wakker PP, Thaler RH, Tversky A (1997). Probabilistic insurance. Journal of Risk and Uncertainty.

[CR46] Zimmer A, Gründl H, Schade CD, Glenzer F (2018). An incentive-compatible experiment on probabilistic insurance and implications for an insurer’s solvency level. Journal of Risk and Insurance.

[CR47] Zimmer A, Schade CD, Gründl H (2009). Is default risk acceptable when purchasing insurance? Experimental evidence for different probability representations, reasons for default, and framings. Journal of Economic Psychology.

