# Supplementary material for: Insurance decisions under nonperformance risk and ambiguity
Source: J Risk Uncertain. 2021 Nov 26;63(3):229–53. doi: 10.1007/s11166-021-09364-7 (PMC8625686; doi:10.1007/s11166-021-09364-7)
Supplement: Supplementary file 1 — Supplementary file1 (PDF 1421 KB) [file 11166_2021_9364_MOESM1_ESM.pdf]

Online Appendix to

"Insurance decisions under nonperformance risk and ambiguity"

Timo R. Lambregts*, Paul van Bruggen† and Han Bleichrodt‡

Journal of Risk and Uncertainty 63(3)

This Online Appendix contains the following five items. First, in Appendix A we provide an overview of all tasks that were presented to participants of our lab experiment. Appendix B gives the instructions as provided at the experiment. Appendix C and Appendix D contain derivations that show how rank-dependent probability weighting and recursive rank-dependent probability weighting respectively cannot accommodate our findings. Lastly, Appendix E provides the results of several additional analyses that are referred to in the main text.

*Erasmus School of Health Policy & Management, Erasmus University Rotterdam, Burgemeester Oudlaan 50, 3062 PA Rotterdam, the Netherlands, and CPB Netherlands Bureau of Economic Policy Analysis. Corresponding author: lambregts@eshpm.eur.nl

†Department of Economics, Tilburg University

‡Erasmus School of Economics, Erasmus University Rotterdam, and Research School of Economics, Australian National University

Appendix A – Experiment tasks

Table 4 Insurance tasks for losses

No insurance				Insurance					
1 – p	0	*p*	$-L$	*1 – p*	$-\pi$	*p*(1 – q)*	$-\pi$	*p*q*	$-\pi - L$
0.9	€0.00	0.1	-€17.50	0.9	-€1.40	0.1*0.8	-€1.40	0.1*0.2	-€18.90
0.8	€0.00	0.2	-€17.50	0.8	-€3.15	0.2*0.9	-€3.15	0.2*0.1	-€20.65
0.7	€0.00	0.3	-€15.00	0.7	-€2.70	0.3*0.6	-€2.70	0.3*0.4	-€17.70
0.6	€0.00	0.4	-€15.00	0.6	-€3.60	0.4*0.6	-€3.60	0.4*0.4	-€18.60
0.5	€0.00	0.5	-€16.00	0.5	-€5.60	0.5*0.7	-€5.60	0.5*0.3	-€21.60
0.4	€0.00	0.6	-€12.50	0.4	-€6.75	0.6*0.9	-€6.75	0.6*0.1	-€19.25
0.3	€0.00	0.7	-€10.00	0.3	-€5.60	0.7*0.8	-€5.60	0.7*0.2	-€15.60
0.2	€0.00	0.8	-€14.00	0.2	-€5.60	0.8*0.5	-€5.60	0.8*0.5	-€19.60
0.1	€0.00	0.9	-€14.00	0.1	-€6.30	0.9*0.5	-€6.30	0.1*0.5	-€20.30
0.8	€0.00	0.2	-€17.50	0.8	-€3.15	0.2*0.9	-€3.15	0.2*0.1	-€20.65

Table 5 Insurance tasks for gains

No insurance				Insurance					
1 – p	0	*p*	G	*1 – p*	π	*p*(1 – q)*	π	*p*q*	$\pi + G$
0.9	€0.00	0.1	€55.00	0.9	€4.40	0.1*0.8	€4.40	0.1*0.2	€59.40
0.7	€0.00	0.3	€65.00	0.7	€11.70	0.3*0.6	€11.70	0.3*0.4	€76.70
0.5	€0.00	0.5	€58.00	0.5	€20.30	0.5*0.7	€20.30	0.5*0.3	€78.30
0.3	€0.00	0.7	€70.00	0.3	€39.20	0.7*0.8	€39.20	0.7*0.2	€109.20
0.1	€0.00	0.9	€40.00	0.1	€18.00	0.9*0.5	€18.00	0.1*0.5	€58.00

Table 6 Risk aversion tasks

Risk averse				Risk seeking			
$1-p$	$-r$	p	$-k$	$1-p$	0	p	$-k-\frac{1-p}{p}r$
0.9	-€1.00	0.1	-€6.00	0.9	€0.00	0.1	-€15.00
0.8	-€3.50	0.2	-€6.00	0.8	€0.00	0.2	-€20.00
0.7	-€3.00	0.3	-€9.00	0.7	€0.00	0.3	-€16.00
0.6	-€8.00	0.4	-€6.00	0.6	€0.00	0.4	-€18.00
0.5	-€10.00	0.5	-€11.00	0.5	€0.00	0.5	-€21.00
0.4	-€6.00	0.6	-€10.00	0.4	€0.00	0.6	-€14.00
0.3	-€3.50	0.7	-€11.00	0.3	€0.00	0.7	-€12.50
0.2	-€10.00	0.8	-€15.00	0.2	€0.00	0.8	-€17.50
0.1	-€9.00	0.9	-€18.00	0.1	€0.00	0.9	-€19.00

Table 7 Risk prudence tasks

Risk prudent			Risk imprudent		
$-x-k$	$-x+s$	$-x-\mathrm{s}$	$-x$	$-x+\mathrm{s}$	$-x-\mathrm{s}$
-€11.00	-€2.00	-€10.00	-€6.00	-€7.00	-€15.00
-€13.00	-€2.00	-€12.00	-€7.00	-€8.00	-€18.00
-€14.00	-€4.00	-€16.00	-€10.00	-€8.00	-€20.00
-€13.00	-€3.00	-€7.00	-€5.00	-€11.00	-€15.00
-€16.00	-€3.00	-€9.00	-€6.00	-€13.00	-€19.00
-€13.00	-€2.00	-€18.00	-€10.00	-€5.00	-€21.00
-€15.00	-€6.00	-€16.00	-€11.00	-€10.00	-€20.00
-€14.00	-€3.00	-€15.00	-€9.00	-€8.00	-€20.00
-€14.00	-€2.00	-€10.00	-€6.00	-€10.00	-€18.00

Notes: Sure amount c has been deducted such that all outcomes are negative. $-x-k$ and $-x+\tilde{\varepsilon}$ both occur with probability 0.5 (with $-x+s$ and $-x-s$ occurring with probability 0.25).

Table 8 Ambiguity aversion tasks

Ambiguity averse				Ambiguity seeking			
$1-p+\tilde{\varepsilon}$	0	$p+\tilde{\varepsilon}$	$-k$	$1-p+\tilde{\varepsilon}$	0	$p+\tilde{\varepsilon}$	$-k$
0.9	€0.00	0.1	-€17.50	0.9	€0.00	0.1	-€17.50
0.8	€0.00	0.2	-€15.00	0.8	€0.00	0.2	-€15.00
0.7	€0.00	0.3	-€16.00	0.7	€0.00	0.3	-€16.00
0.6	€0.00	0.4	-€10.00	0.6	€0.00	0.4	-€10.00
0.5	€0.00	0.5	-€18.00	0.5	€0.00	0.5	-€18.00
0.4	€0.00	0.6	-€21.00	0.4	€0.00	0.6	-€21.00
0.3	€0.00	0.7	-€12.50	0.3	€0.00	0.7	-€12.50
0.2	€0.00	0.8	-€19.00	0.2	€0.00	0.8	-€19.00
0.1	€0.00	0.9	-€20.00	0.1	€0.00	0.9	-€20.00

Table 9 Ambiguity prudence tasks

Ambiguity prudent				Ambiguity imprudent			
$p+k$	$q-\varepsilon$	$q+\varepsilon$	$-L$	p	$q-\varepsilon+k$	$q+\varepsilon+k$	$-L$
0.9	0.1	0.6	-€17.00	0.5	0.5	1.0	-€17.00
0.8	0.2	0.7	-€15.00	0.5	0.5	1.0	-€15.00
0.7	0.3	0.8	-€14.00	0.5	0.5	1.0	-€14.00
0.8	0.2	0.8	-€13.00	0.6	0.4	1.0	-€13.00
0.7	0.3	0.7	-€11.00	0.4	0.6	1.0	-€11.00

Note: $q-\varepsilon$ and $q+\varepsilon$ are the lower and upper bound of the uncertainty interval $q+\tilde{\varepsilon}$.

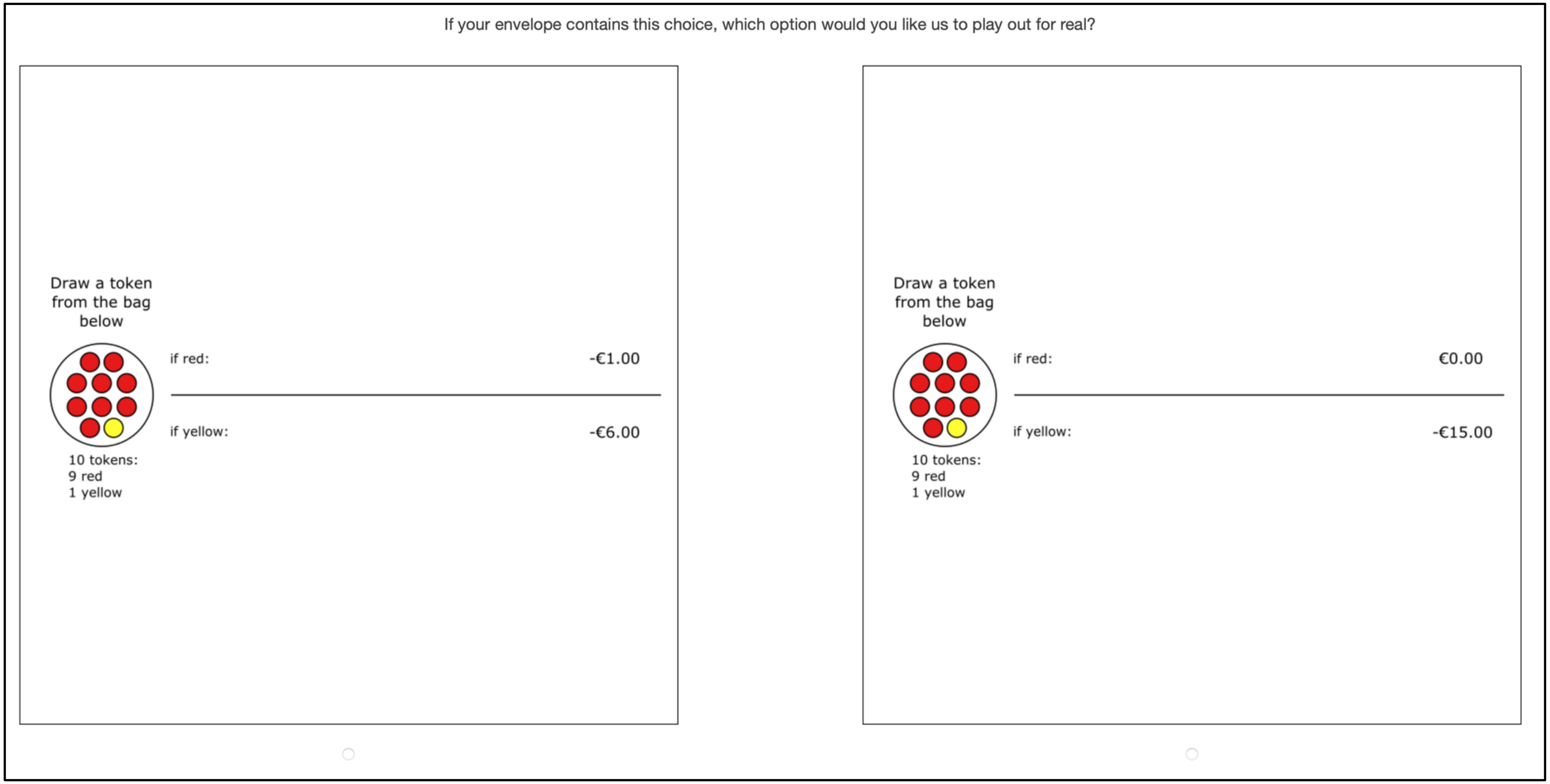

Figure 10 Example of a risk aversion task

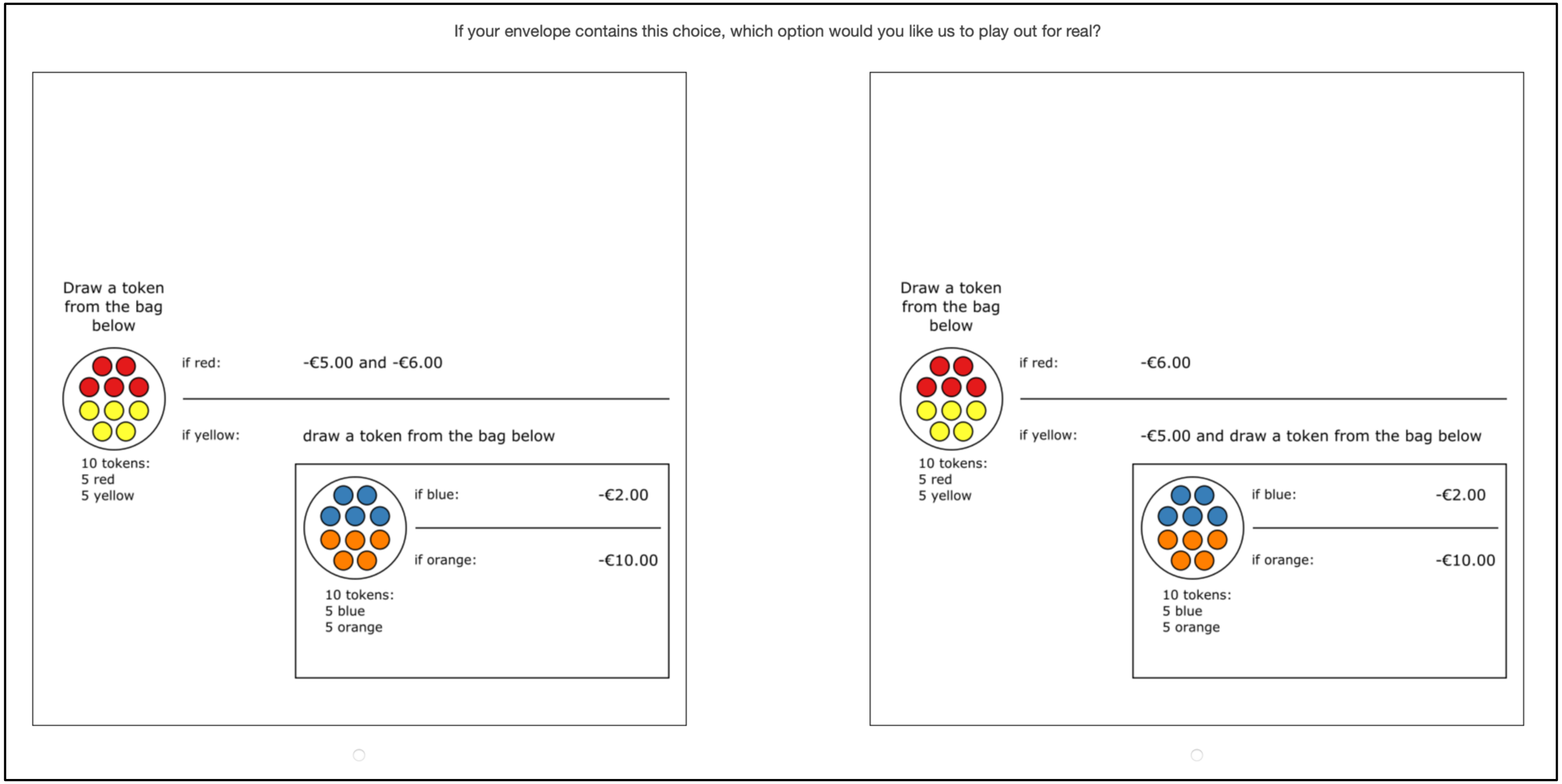

Figure 11 Example of a risk prudence task

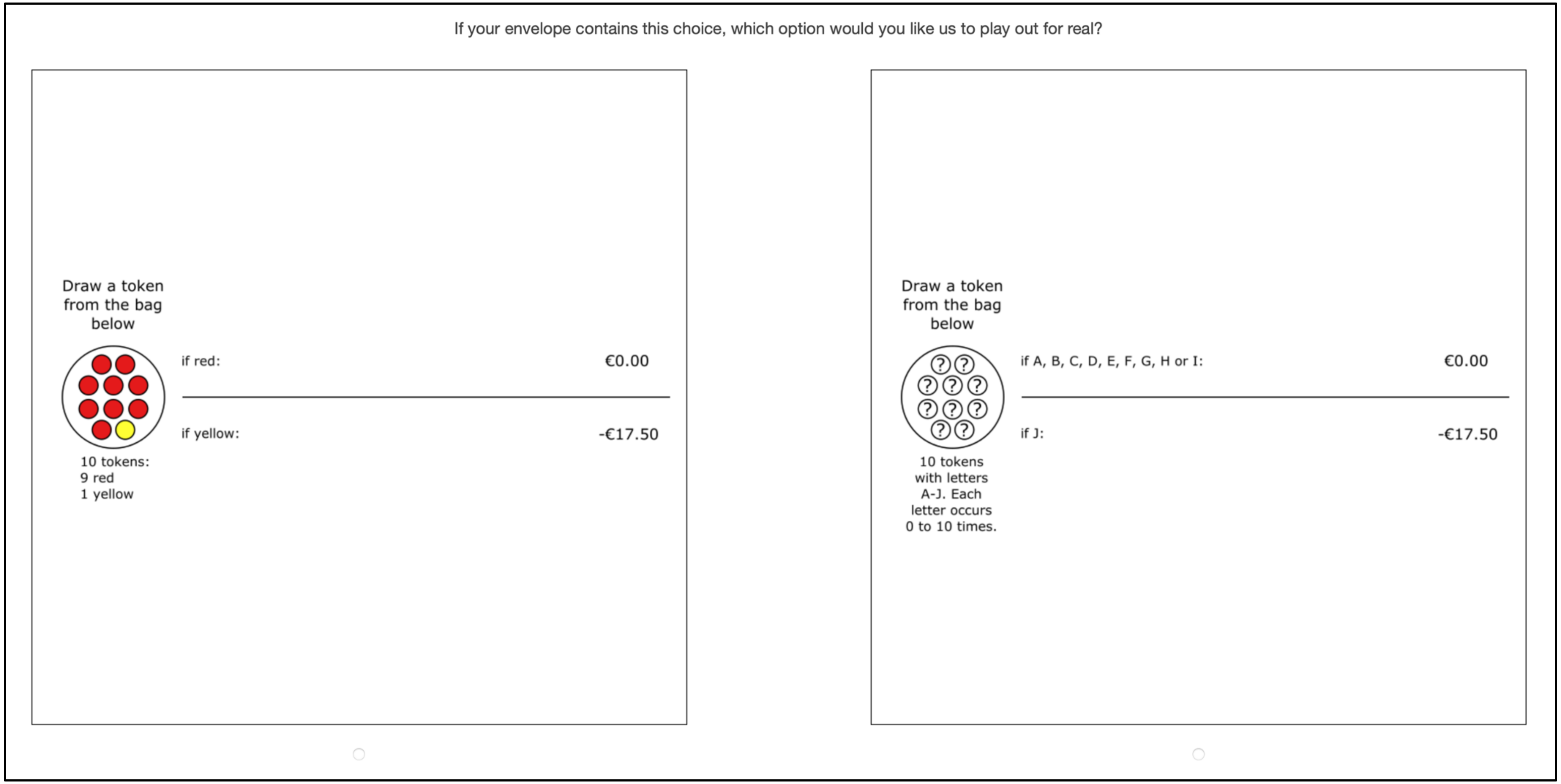

Figure 12 Example of an ambiguity aversion task

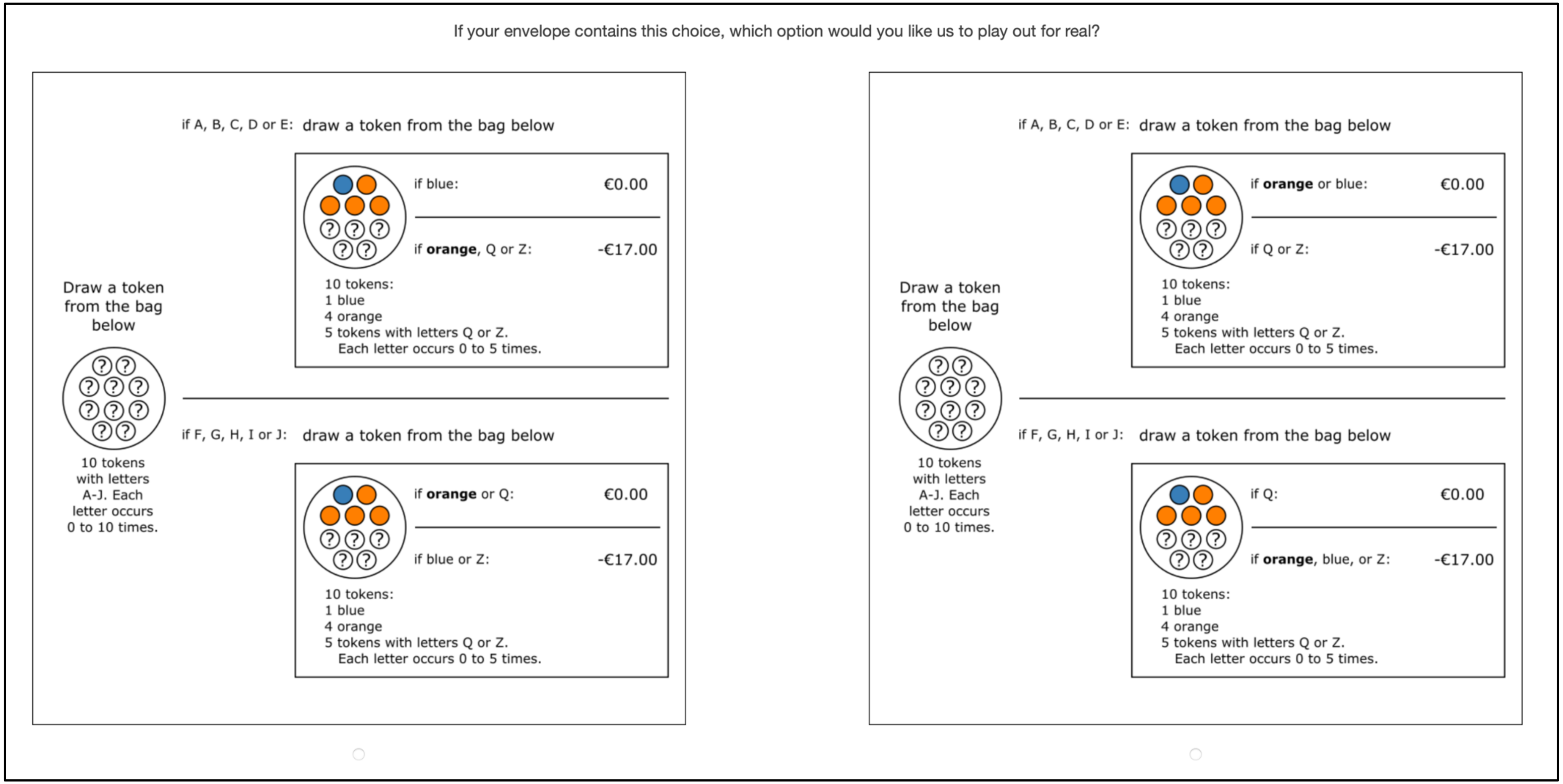

Figure 13 Example of an ambiguity prudence task

Appendix B – Instructions

B1 Instructions before entering the cubicles

Welcome all and thank you for participating.

You will be asked to make 92 choices between two options involving monetary outcomes. Before starting the experiment, you receive €25. If you choose not to finish the experiment you will have to return this money. Before starting, you will also select one of 92 closed envelopes. You are not allowed to open the envelope. Each sealed envelope represents one of the decision tasks. The decision task that envelope you have selected contains, will be played out for real after you have finished all choice tasks. The outcomes of the decision tasks range from -€21.00 to +€109.20.

The experiment will start with an explanation of the decision tasks. If you have any questions, one of us will be available in the control room between the cubicles. If you have finished the experiment, you can report back to the control room and you will be asked to wait in your cubicle until everyone has finished.

You can now – one by one – collect your €25, select an envelope. We will also number your envelope. You can enter this number in the first question. When everyone has received money and an envelope, we will walk to the cubicles.

B2 Instructions in the cubicles before the experiment

General

- You will be asked to make **92** choices between two options involving monetary outcomes under risk and uncertainty.
- These questions are divided over **12** parts, all of which start with **1** practice question.
- Additionally, you will be asked to answer **4** background questions.
- After the instructions, you will be asked to answer **5** practice questions.
- Except for the practice questions, there are no 'right' or 'wrong' answers. We are only interested in your preferences.
- You can withdraw at any time. By withdrawing you forego the entitlement to any compensation; you will then have to return the €25.00 show-up fee.

Compensation

- Compensation consists of a show-up fee and a variable pay.
- You have received a show-up fee of €25.00, conditional on completing all 92 choices and 4 background questions.
- The variable pay will be determined as follows:
- You have drawn a sealed envelope containing one of these 92 choices (a number from 1 to 92).
- Thus each choice has an equal chance to be selected.
- Your envelope will be opened when everybody has finished all choices and background questions. Thus, if you finish early, you might have to wait for the others to finish.
- The option that you have chosen in that particular choice will then be played out and paid for real.
- The possible outcomes of the variable pay range from -€21.60 to +€109.20 (with an average of €0.00) in addition to the show-up fee of €25.00.

Rules

- Talking, eating or drinking anything other than water is not allowed.
- Please turn off your cell phone now.
- Whenever you have a question, please raise your hand. A member of staff will answer your question in private.

Choice tasks

- You will be asked to make choices between two options.
- All options require drawing a token from one or two bags.
- All bags contain 10 tokens of one or more colors or letters.
- A legend is shown next to each bag, stating for each possible token in that bag what the consequence are when that token is drawn. This can be:
    - losing money;
    - gaining money;
    - neither losing nor gaining money; and
    - drawing a token from another bag.
- You can change your response to a previous question within the same part by returning to that question using the 'back' button.

Example

If you draw a yellow token, when playing out the option below:

- if you have chosen the option on the left-hand side, you lose €10.00.
- if you have chosen the option on the right-hand side, you lose €2.00 and you have to draw a token from another bag with blue and orange tokens. Then:
    - if you draw a blue token, you lose nothing in addition to your loss of €2.00. Hence, you lose €2.00 in total.
    - if you draw an orange token, you lose €10.00 in addition to your loss of €2.00. Hence, you lose €12.00 in total.

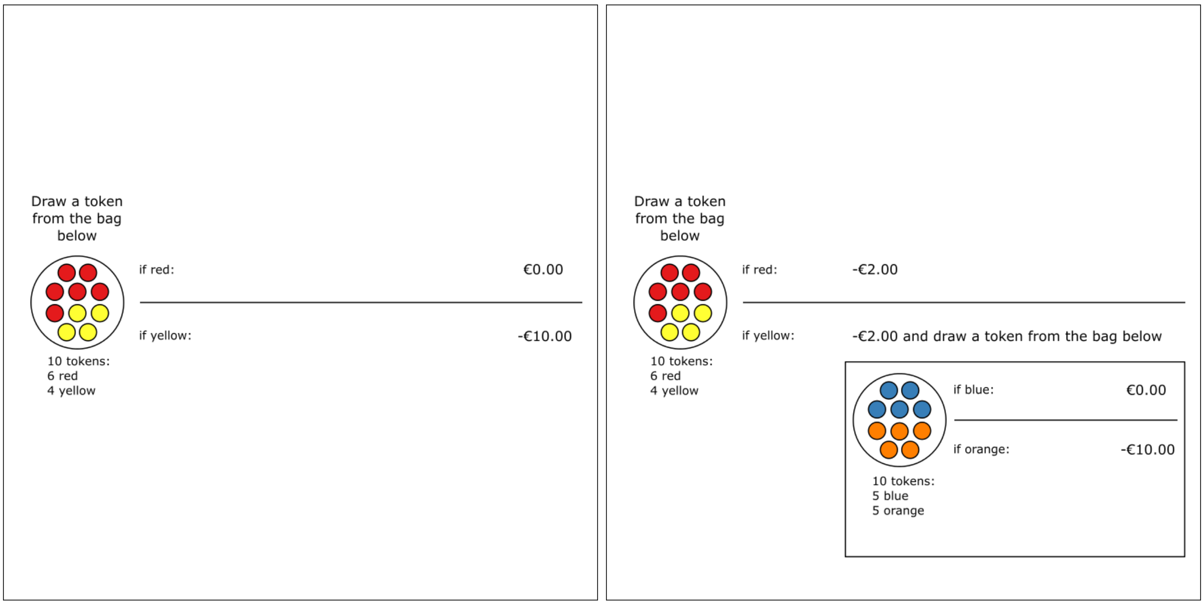

Uncertainty

- Some bags are depicted with 10 question-marked tokens. We call these "bags of unknown composition".
- "Bags of unknown composition" contain 10 tokens with letters. Each letter occurs 0 to 10 times.
- These bags have been randomly filled with 10 tokens before the start of this session, based on the instructions of someone not affiliated with this research and who does not know the purpose of these bags.
- These letters can be any combination of the letters A-J or the letters Q-Z.
- Whether "bags of unknown composition" contain combinations of the letters A-J or of the letters Q-Z is shown in the legend underneath the bags.
- After the practice questions, you will be asked to rank the letters (A-J or J-A and Q-Z or Z-Q).
- This ranking will be used to play out for real the choice task that your envelope contains.

Example

If you draw a token with the letter H, when playing out the option below:

- if you have chosen the option on the left-hand side, you lose €10.00.
- if you have chosen the option on the right-hand side, you lose €2.00 and draw a token from another bag with blue and orange tokens. Then:
    - if you draw a blue token, you lose nothing in addition to your loss of €2.00. Hence, you lose €2.00 in total.
    - if you draw an orange token, you lose €10.00 in addition to your loss of €2.00. Hence, you lose €12.00 in total.

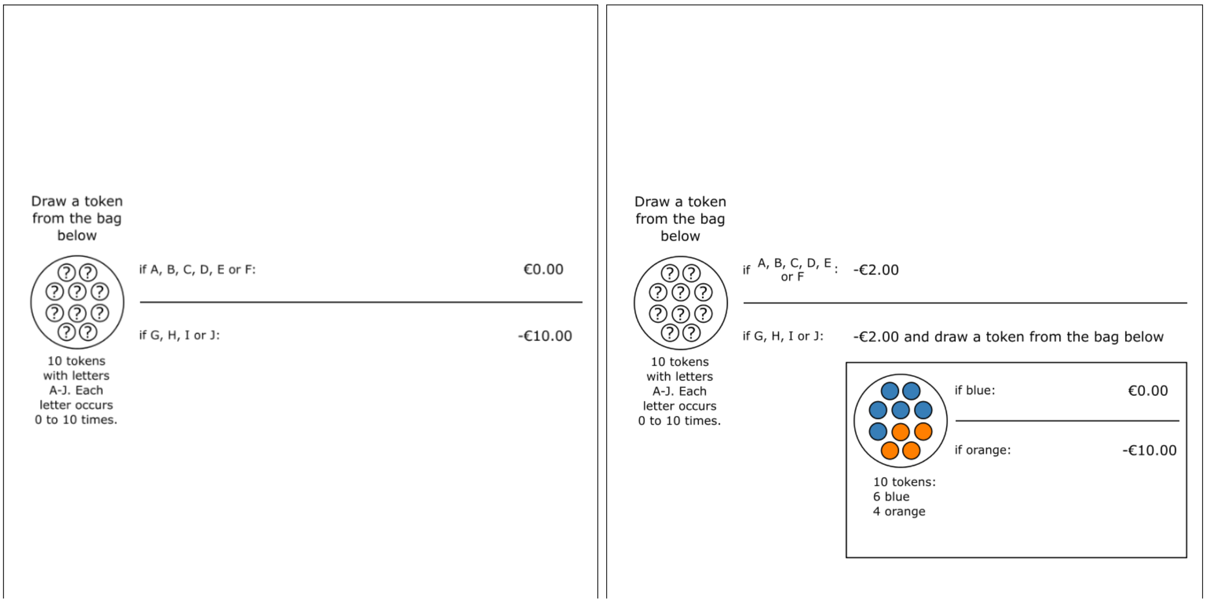

Appendix C – Probability weighting

Under linear utility, which is commonly observed for choices involving only small stakes, no single probability weighting function can explain the majority choices that we observe in our risk aversion and insurance tasks under rank-dependent utility. To illustrate this, let's first look at our risk aversion tasks. Under rank-dependent utility we can write the observed majority choice for $p = 0.1$ as:

$$w(0.1) \cdot (-15) + (1 - w(0.1)) \cdot 0 < w(0.1) \cdot (-6) + (1 - w(0.1)) \cdot (-1).$$

The inequality sign here implies that most of our respondents chose the risk averse option. We can simplify this to $w(0.1) > 0.1$, which implies that most respondents overweighed this probability. Similarly, we find the following implied probability weights for our other risk aversion tasks:

$$w(0.1) > 0.1$$
$$w(0.2) > 0.2$$
$$w(0.3) > 0.3$$
$$w(0.4) > 0.4$$
$$w(0.5) > 0.5$$
$$w(0.6) \sim 0.6$$
$$w(0.7) < 0.7$$
$$w(0.8) < 0.8$$
$$w(0.9) < 0.9$$

Hence, we find probability overweighting for $0.1 \leq p \leq 0.5$ and underweighting for $0.7 \leq p \leq 0.9$. This pattern is consistent with inverse S-shaped probability weighing, which predicts overweighting of small probabilities and underweighting of large probabilities.

Let's next consider the insurance tasks. Under rank-dependent utility with loss ranks we can write the observed majority choice for $p = 0.1$ as:

$$w(0.1) \cdot (-17.5) + (1 - w(0.1)) \cdot 0 > w(0.02) \cdot (-18.9) + (1 - w(0.02)) \cdot (-1.4).$$

We can simplify this to $w(0.1) < w(0.02) + 0.08$. Similarly, we find the following implied probability weights for our other insurance tasks:

$$w(0.1) < w(0.02) + 0.08$$
$$w(0.2) \sim w(0.02) + 0.18$$
$$w(0.3) < w(0.12) + 0.18$$
$$w(0.4) > w(0.16) + 0.24$$
$$w(0.5) > w(0.15) + 0.35$$
$$w(0.6) > w(0.06) + 0.54$$
$$w(0.7) > w(0.14) + 0.56$$
$$w(0.8) > w(0.40) + 0.40$$
$$w(0.9) > w(0.45) + 0.45$$

Note that the numbers on the right of the inequality signs sum to the number on the left. The final three inequalities show that the weights of probabilities 0.7, 0.8 and 0.9 (on the left-hand side) must be overweighed, as they must be greater than (on the right-hand side) the sum of a linear probability weight and the weights of probabilities smaller than 0.5, and the latter were found to be overweighed in the risk aversion tasks. Yet the probabilities bigger than 0.7 were found to be underweighted in the risk aversion tasks, a contradiction.

Hence, rank dependent utility with linear utility cannot explain our majority choices.

Appendix D – Recursive rank dependent utility

The reason why probability weighting (in the form of rank dependent utility) cannot capture our findings is that for the risk aversion tasks, we find risk aversion for probabilities (of the worst outcome) between 0.1 and 0.5 and risk loving choices for probabilities between 0.6 and 0.9, which indicates inverse S-shaped probability weighting. Such inverse S probability weighting implies that insurance should be attractive when the insurable risk is small. Yet, subjects, on average, chose not to buy insurance for the probabilities, and did for larger insurable risks. One possible explanation for this apparent contradiction is that subjects do weigh probabilities but violate the reduction of compound lotteries axiom. After all, risk aversion was measured with a single-stage task, whereas the insurance option involves two stages. However, as we show in this Appendix, allowing for such violations does not help to accommodate our findings.

Recursive rank dependent utility (as applied by e.g., Freeman (2017)) is a form of probability weighting that allows for violations of the reduction of compound lotteries axiom when applying probability weighting. It works by first evaluating the second stage according to rank dependent utility with some probability weighting function $w(p)$, calculating the certainty equivalent of the second stage, and then substituting this certainty equivalent of the second stage in the first stage. The first stage is then evaluated according to rank dependent utility, using the same probability weighting function $w(p)$ as in evaluating the second stage, which means the procedure satisfies Segal's (1990) time neutrality axiom.

A first observation is that, for a given probability weighting function, the evaluation of the risk aversion tasks and the no insurance option are the same for rank dependent utility and its recursive form, because they involve only one stage. Therefore, the choices for the risk aversion tasks still indicate inverse S probability weighting. As it turns out, the insurance option is even more attractive for small insurable risks under recursive rank dependent utility than under rank dependent utility with inverse S probability weighting.

To see this, we can write out the utility representation of the insurance option under rank dependent utility:

$$w(pq)(-\pi - L) + [1 - w(pq)](-\pi).$$

Under recursive rank dependent utility, the value of the second stage is

$$w(q)(-\pi - L) + [1 - w(q)](-\pi)$$

and substituting this into the representation of the first stage, the value of the insurance option is

$$w(p)[w(q)(-\pi - L) + [1 - w(q)](-\pi)] + [1 - w(p)](-\pi).$$

Thus, under rank dependent utility the weight on the best outcome, $-\pi$, is

$$[1 - w(q)]$$

whereas under recursive rank dependent utility it is

$$[1 - w(p)w(q)]$$

which is obviously bigger. Thus, under recursive rank dependent utility, the best outcome of the insurance option is weighted more strongly than under rank dependent utility, which makes insurance more attractive. Given that the difficulty was with accommodating the distaste for some of the insurance options, recursive rank dependent utility cannot accommodate this.

References

Freeman, D. (2017), Risk taking with background risk under recursive rank-dependent utility, *Mathematical Social Sciences, 87*, 72-74. doi.org/10.1016/j.mathsocsci.2017.03.003

Segal, U. (1990). Two-stage lotteries without the reduction axiom. *Econometrica, 58*(2), 349-377. doi.org/10.2307/2938207

Appendix E – Supplementary analyses

Table 10 Probit regression with the number of ambiguity averse and prudent choices

	Choose insurance	Average marginal effect
KU	-0.13**	-0.05
	(0.05)	(0.02)
UK	-0.06	-0.02
	(0.06)	(0.02)
UU	-0.20***	-0.07
	(0.06)	(0.02)
Risk aversion	0.07***	0.03
	(0.03)	(0.01)
Risk prudence	-0.05**	-0.02
	(0.02)	(001)
p	1.11***	0.39
	(0.18)	(0.06)
Ambiguity aversion	0.04*	0.02
	(0.03)	(0.01)
Ambiguity prudence	0.00	0.000
	(0.03)	(0.01)
Male	0.22**	0.08
	(0.10)	(0.04)
Dutch	0.05	0.02
	(010)	(0.04)
Constant	-0.55**	
	(0.25)	

Notes: N = 4,176. Asterisks indicate a p-value < 0.05 (**) and < 0.01 (***). Robust standard errors between parentheses and clustered by subject. *KU*, *UK* and *UU* are treatment dummies indicating whether the insurable risk and nonperformance risk respectively are known (K) or unknown (U). Risk aversion, risk prudence and ambiguity aversion are the number of risk averse, risk prudent and ambiguity averse choices (out of nine). Ambiguity prudence is the number of ambiguity prudent choices (out of five). p is the insurable risk and can take any decimal value from 0.1 till 0.9.

Table 11 Probit regression interacting risk aversion with a large insurable risk

	Choose insurance	Average marginal effect
KU	-0.14**	-0.05
	(0.05)	(0.02)
UK	-0.06	-0.02
	(0.06)	(0.02)
UU	-0.20***	-0.07
	(0.06)	(0.02)
Risk aversion	0.13***	0.04
	(0.03)	(0.01)
Risk prudence	-0.04**	-0.02
	(0.02)	(0.01)
$p \geq 0.5$	1.08***	0.38
	(0.20)	(0.06)
Risk aversion $\times$ $p \geq 0.5$	-0.11***	-0.04
	(0.04)	(0.01)
Male	0.23**	0.08
	(0.10)	(0.04)
Dutch	0.05	0.02
	(0.10)	(0.04)
Constant	-0.37	
	(0.20)	

Notes: N = 4,176. Asterisks indicate a p-value < 0.05 (**) and < 0.01 (***). Robust standard errors between parentheses and clustered by subject. KU, UK and UU are treatment dummies indicating whether the insurable risk and nonperformance risk respectively are known (K) or unknown (U). Risk aversion and risk prudence are the number of risk averse and risk prudent choices (out of nine). p is the insurable risk and can take any decimal value from 0.1 till 0.9.

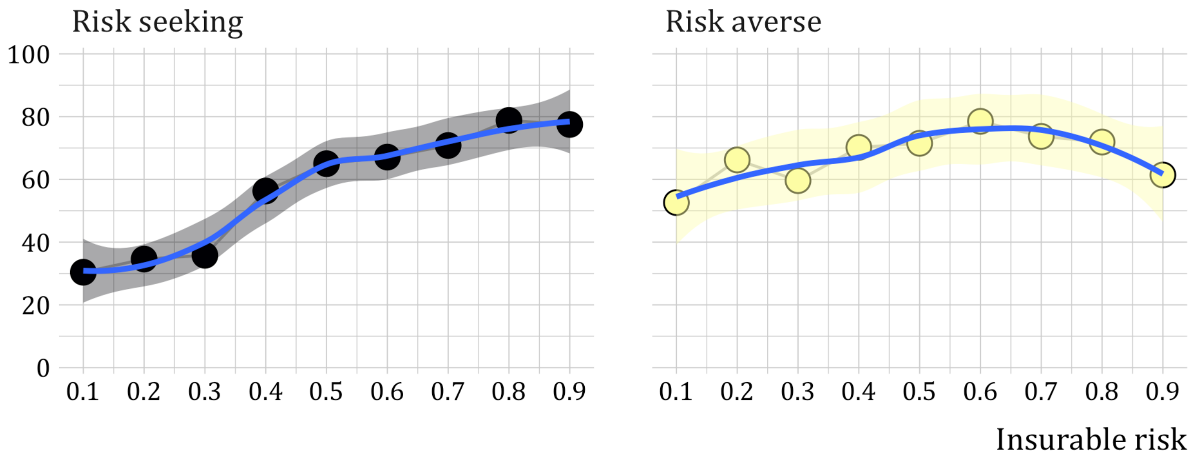

Figure 14 Proportion of insurance choices for more risk seeking and risk averse individuals by insurable risk

Notes: Risk seeking means less than 5 risk averse choices. Risk averse means more than 4 risk averse choices. Trend line fitted by loess method. Bands show the 95% confidence interval.
